# Sub-kJ/mol Precision at Practical Cost for Molecular Solids through Local Coupled-Cluster Approaches

**DOI:** 10.1021/acs.jpca.6c05975

**Published:** 2026-10-14

**Authors:** Khanh Ngoc Pham, Marcin Modrzejewski, Péter R. Nagy, Jiří Klimeš

**Affiliations:** † Department of Chemical Physics and Optics, Faculty of Mathematics and Physics, 138735 Charles University, Ke Karlovu 3, Prague 2 CZ-12116, Czech Republic; ‡ Faculty of Chemistry, 49605 University of Warsaw, Pasteura 1, Warsaw 02-093, Poland; § Department of Physical Chemistry and Materials Science, Faculty of Chemical Technology and Biotechnology, 61810 Budapest University of Technology and Economics, Műegyetem rkp. 3., Budapest H-1111, Hungary; ∥ HUN-REN−BME Quantum Chemistry Research Group, Műegyetem rkp. 3., Budapest H-1111, Hungary; ⊥ MTA−BME Lendület Quantum Chemistry Research Group, Muegyetem rkp. 3., Budapest H-1111, Hungary

## Abstract

Generation of numerically converged coupled-cluster reference energies of molecular crystals remains highly non-routine. Yet, such data points are critically needed as computational methodologies strive to reach correlated wave-function accuracy in condensed-phase systems. Localized-orbital coupled-cluster approaches applied within the many-body expansion promise a scalable route to these reference results. Here, we report solutions to a technical challenge when using this methodology—at long intermolecular distances, the numerical noise due to local approximations can accumulate for some molecular crystals into a large-scale drift of the correlation energy, especially in the nonadditive three-body contributions. Within the local natural orbital (LNO) coupled-cluster method, we suggest well-balanced approaches to overcome this issue. At the benchmark level, we propose more accurate LNO-CCSD­(T) settings that remove the artifacts at long distances and attain a numerical precision of 0.2 kJ/mol (maximum error) in the lattice energy, with a practical cost that is orders of magnitude lower than that of canonical CCSD­(T). As an even more scalable solution, we propose a multi-level strategy that utilizes the established routine LNO-CCSD­(T) precision tiers (Tight, vTight) but retains the numerical stability of the benchmark level by treating long-distance three-body systems with the random-phase approximation (RPA).

## Graphical abstract

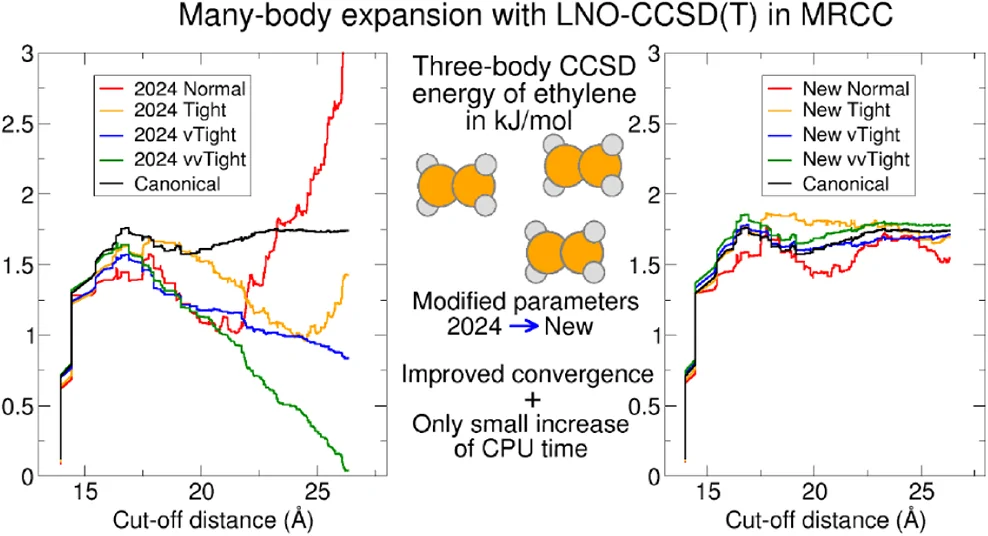

## 1 Introduction

The computational modeling of organic molecular crystals is a rapidly evolving field of computational chemistry with developments driven by possible industrial applications, e.g., in the prediction of bioavailability of pharmaceuticals.^1–9^ However, the pace of development of approximate quantum-chemical methods is limited by scarcity of high-quality data. For example, chemically accurate static lattice energies, at a correlated ab initio level of theory, are available for only about 30 systems.^10–15^ One promising strategy to fill this gap is the fragment-based wave-function approach, pioneered for solids by Stoll^16–19^ and others.^20^ In the context of molecular crystals the method has morphed into the systematically improvable many-body expansion (MBE) of the lattice energy.^2,10,12,15,21–31^ The use of MBE gives access not only to the total energy, but also reveals the constituent pairwise and nonadditive interaction energies, which provide additional validation data for approximate methods.^32–40^ Nonetheless, generating a reliable MBE requires coupled-cluster wave-function theory with singles, doubles, and perturbative triples (CCSD­(T)). Any simpler wave-function method, such as Møller-Plesset approximations and random-phase approximation, yields systematic errors either in dimers or in the nonadditive contributions of many-body clusters.^37,38,40–42^

Identifying an efficient method that can match the accuracy of canonical CCSD­(T) at the 0.1 kJ/mol scale for both the pairwise and nonadditive parts of MBE is up to now an unsolved problem, but, with the current generation of localized-orbital coupled-cluster approaches, a method of this kind appears to be a possible solution. Here, we utilize some of the most developed implementations of the local correlation approaches:^43^ the domain-based local pair natural orbital^44,45^ (DLPNO), pair natural orbital local^46^ (PNO-L), and localized natural orbital^43,47–49^ (LNO) schemes. These methods can, depending on the problem size, reduce the computational cost by orders of magnitude relative to the canonical coupled-cluster implementation. They are widely used to obtain interaction energies for systems ranging from small dimers to large molecular complexes.^43,50–60^ Furthermore, they provide a scalable route to the beyond density functional theory (DFT) data required by the next generation of machine learning interatomic potentials (MLIPs). For example, the local coupled-cluster approach was recently showcased as an efficient ground-truth method in a MLIP-based simulation of the structural and transport properties in liquid water.^61^

The MBE treatment of molecular crystals with a local coupled cluster approximation is, however, a property not yet covered broadly enough by previous benchmarks (see review in ref 43), and one that requires careful testing. The application to MBE is not trivial for the following two main reasons.

i The ordering of crystal phases is controlled by small energy differences.^62^ This makes the approximate results sensitive to the treatment of long-range dispersion interactions.^63,64^

ii The many-body expansion requires extremely tight thresholds to retain numerical stability, e.g., for the self-consistent field,^65,66^ amplitude equations, and possibly other method-specific steps.

Therefore, thorough tests are needed to establish the accuracy-cost trade-offs of localized-orbital approximations in coupled-cluster theory for molecular crystals.

In this work, we assess PNO-LCCSD­(T), DLPNO-CCSD(T), and LNO-CCSD­(T) for their ability to describe the two-body and three-body binding-energy contributions of molecular solids exhibiting diverse intermolecular interactions. The assessment is carried out for systems where high-level reference CCSD­(T) data are available, including solid ethane, ethylene, acetylene, ammonia, methanol, and formic acid crystals.^37,40^ Using these reference data for comparison, we first analyze the effects of various thresholds on convergence of the results with the basis-set size and with the cutoff distance. We then evaluate the accuracy of the local correlation approaches using different threshold settings. Finally, we suggest improved LNO-CCSD­(T) settings and make recommendations for two computational protocols: a high-precision benchmark approach (0.2 kJ/mol maximum error) and a more affordable composite strategy scalable to large systems.

## 2 Methods

### 2.1 Many-Body Expansion

In the MBE approach, the total energy *E* of an *N*-monomer system,

1 $$E=\overset{N}{\underset{i=1}{\sum }}E_{i}+\overset{N}{\underset{i<j}{\sum }}\Delta E_{i,j}+\overset{N}{\underset{i<j<k}{\sum }}\Delta E_{i,j,k}+\cdot \cdot \cdot$$

is resolved into a hierarchy of *n*-body contributions: the monomer energies, *E*_*i*_, the pairwise interactions,

2 $$\Delta E_{i,j}=E_{i,j}-E_{i}-E_{j}$$

and the nonadditive interactions, with the leading three-body contribution

3 $$\Delta E_{i,j,k}=E_{i,j,k}-\Delta E_{i,j}-\Delta E_{i,k}-\Delta E_{j,k}-E_{i}-E_{j}-E_{k}$$

We consider only structures with a single molecule in the asymmetric unit, defining it as the reference (ref) molecule. Furthermore, we assume that the geometry of the molecule in gas phase is identical to its geometry in solid. Under these conditions, the crystal lattice energy per molecule, *E*_latt_, can be expressed as

4 $$E_{\mathrm{latt}}=\frac{1}{2}\underset{j}{\sum }\Delta E_{ref,j}+\frac{1}{3}\underset{j<k}{\sum }\Delta E_{ref,j,k}+\cdot \cdot \cdot$$

The contributions higher than three-body are non-negligible,^37,40^ but their calculation is hindered by errors due to finite precision.^65^ A feasible way to account for those physical contributions is via periodic embedding, for example, using a Hartree-Fock (HF) implementation with periodic boundary conditions (PBC), as in refs 12,15 or 31 Therefore, in this work, the explicit many-body expansion is limited to the two- and three-body terms and supplemented by the periodic HF correction,

5 $$\Delta E^{\mathrm{HF}}\left(\mathrm{PBC}\right)=E^{\mathrm{HF}}\left(\mathrm{PBC}\right)-E^{\mathrm{HF}}\left(\mathrm{MBE}\right)$$

In practical applications, the summations appearing in eq 4 are truncated at finite distance. For dimers, the distance is defined as the average Cartesian distance between all atom pairs belonging to the two molecules. For trimers, the distance is defined as the sum of the distances of all dimers forming the cluster. The convergence behavior of the two-body and three-body energies with respect to this cutoff has been extensively analyzed for all systems considered in our previous works.^37,40^ On the basis of those convergence studies, we adopt here the same cutoff distances for the two-body and three-body terms as established previously. The number of dimers calculated for all considered systems as a function of the cutoff distance is shown in Figure S1 of Supporting Information.

### 2.2 Precision Control of Localized-Orbital Approaches

The accuracy of localized-orbital coupled-cluster methods depends on several systematically improvable approximations. The corresponding thresholds are typically arranged into predefined sets defining different accuracy tiers, which are valid for various practical use cases. In this work, we systematically examine how these thresholds influence the accuracy of the calculated many-body contributions.

For the PNO-LCCSD­(T) method, we employed the Default, Tight, and vTight settings,^46^ controlled by the domain approximation (DOMOPT) and pair approximation (PAIROPT) options, as summarized in Table S1 of Supporting Information. Previous studies have shown that the Default pair approximation is generally accurate, and the PAIROPT = TIGHT setting is typically used for challenging, extended systems.^46,58,67^ Nevertheless, the cumulative effect of a large number of fragment contributions in the *n*-body terms obtained from MBE calculations may require a tighter pair approximation. To examine this possibility, we performed tests for ethylene and ammonia, with the results summarized in Table S4 of Supporting Information. The obtained results show that the energy deviations between the Default and Tight pair approximations are relatively small, amounting to at most 0.3 kJ/mol for the two-body contributions of ethylene and only 0.1 kJ/mol for the three-body contributions of ammonia. Despite these small deviations, we used the Tight pair approximation in this study to ensure that the *n*-body contributions are determined with sufficient accuracy, while varying only the domain-approximation settings.

For DLPNO-CCSD­(T), we used the LoosePNO, NormalPNO, and TightPNO thresholds,^44,68^ listed in Table S2 of Supporting Information. Finally, for the LNO-CCSD­(T) method, we applied the Normal, Tight, vTight, and vvTight settings,^49^ defined by a set of cutoffs summarized in Table S3 of Supporting Information.

It is important to note that the PNO-L, DLPNO, and LNO methods employ partially similar but not identical approximations and their similarly named threshold combinations not necessarily translate into identical performance. Nevertheless, practical experience so far shows that systematic improvement can be expected in general with all three methods upon tightening their settings bundled into these composite threshold combinations.^43^ The numerical performance of these thresholds is analyzed in detail in Section 3.

### 2.3 Technical Details

All explicit MBE calculations employ Dunning’s augmented correlation-consistent basis sets^69^ with the counterpoise correction applied within each molecular cluster. The basis set names are shortened as AV*X*Z with *X* ∈ T, Q. The two-body correlation energies are extrapolated to the complete basis-set (CBS) limit using the two-point formula of Halkier et al.^70^ The three-body nonadditive energies are taken directly from the AVTZ calculations without extrapolation, as these terms exhibit much faster basis-set convergence than the two-body components.^37,40,71^

The canonical CCSD­(T) results presented here are taken from refs 37 and 40, where they were obtained using the Molpro program.^72^ The canonical calculations involved extremely tight self-consistent-field (SCF) and amplitude equations controlled by, respectively, the SCF keyword energy = 1 × 10^–12^ and the amplitude threshold 1 × 10^–8^. The PNO-LCCSD­(T) calculations were carried out in Molpro 2025.1 using SCF settings identical to the canonical calculations. The DLPNO-CCSD­(T) calculations were conducted in the ORCA 6.0.1 program^73^ with the SCF step controlled by TolE = 1 × 10^–9^ . The LNO-CCSD­(T) and LMP2 calculations were performed using Mrcc version 2025.1.0,^74–76^ with SCF controlled by scfdtol = 8. The RPA+ph energies were generated using the beyond-rpa program^77^ with accuracy default.

The periodic DFT and HF lattice energies were calculated using VASP^78,79^ with sufficiently dense *k*-point meshes and large simulation cells to ensure converged results (Table S6 of Supporting Information). The Coulomb cutoff technique (HFRCUT tag in VASP)^80,81^ was employed to accelerate the convergence of the Fock exchange. All calculations employed hard PAW potentials and a plane-wave energy cutoff of 1500 eV.

## 3 Results

### 3.1 Performance and Challenges with Off-the-Shelf Settings

The accumulation of thousands of small energy contributions is typically needed to reach the limit of converged lattice energy within a few tenths of a kJ/mol/molecule, that is, with a precision necessary to reliably resolve polymorph energy differences.^62^ This requires summing the terms at large intermolecular distances and sufficiently converging to the complete basis-set limit. Here we assess the local approximations on two- and three-body contributions and their basis set dependence via comparison against the canonical CCSD(T) data computed for ethane, ethylene, acetylene, ammonia, methanol, and formic acid.

#### 3.1.1 Distance Convergence

For each examined method, the distance-dependent convergence of the *n*-body contributions is found to be largely consistent across all systems considered. We therefore present in the main text only a selection of examples that illustrate large issues, while the results for the remaining systems are provided in Supporting Information and referenced where appropriate.

For the two-body energies, in most cases the local coupled-cluster correlation energies are reasonably parallel to the canonical CCSD­(T) curve and correctly reproduce its plateau at large molecule–molecule separations (Figure 1). However, there are exceptions where the total two-body contribution does not saturate with the increasing cutoff distance in the local coupled-cluster approximations:

i PNO-LCCSD­(T)/Default exhibits a divergence for acetylene (Figure 1a) and ammonia (Figure S4c in Supporting Information).
ii DLPNO-CCSD­(T)/Normal exhibits a drift in formic acid (Figure 1b).
iii LNO-CCSD­(T) does not converge towards the reference for ethylene (Figure 1c).
iv LNO-CCSD­(T)/Tight shows a drift that levels off at larger cutoff distance in ammonia (Figure 1d).

**Figure 1:**
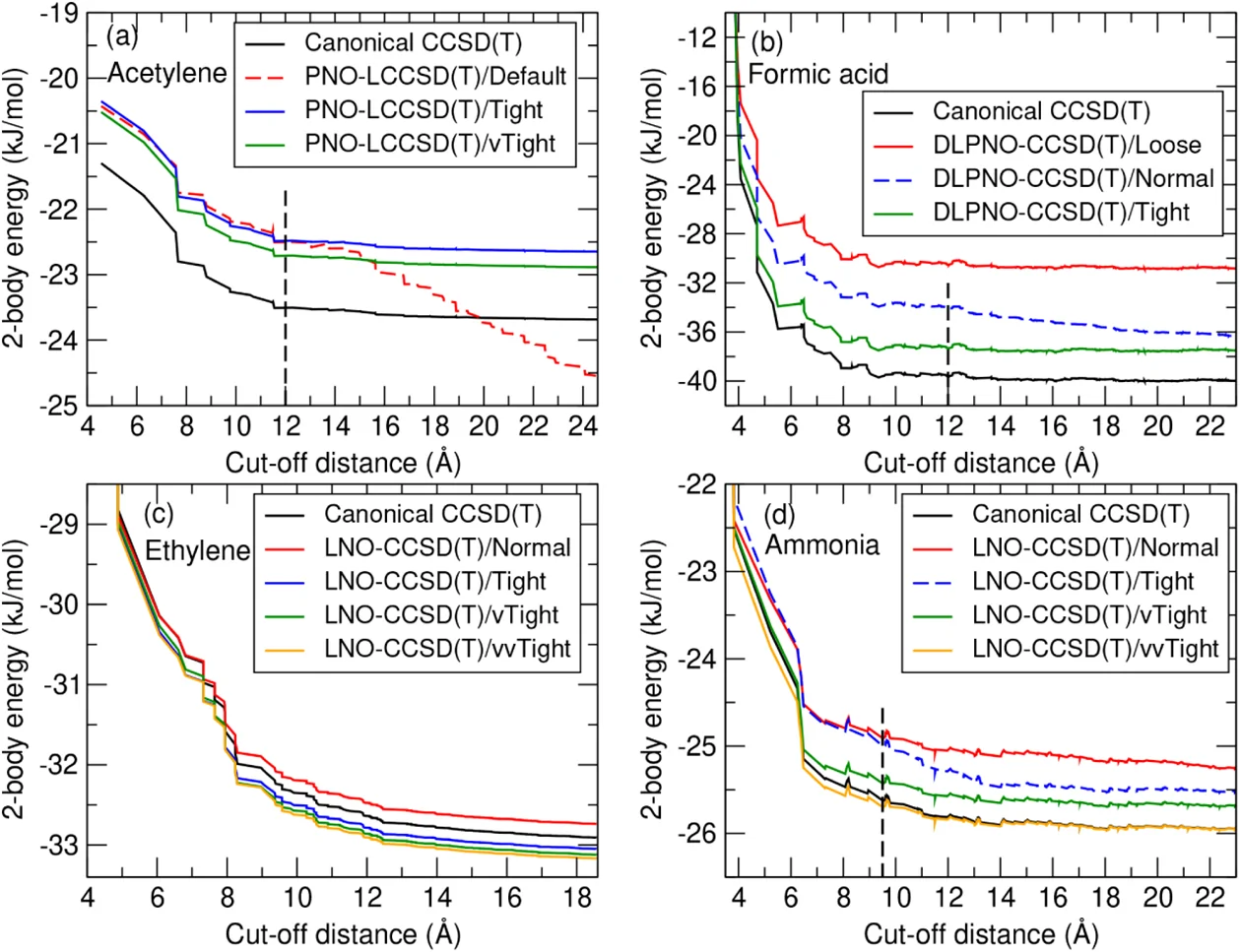
Distance convergence of the correlation contribution to the two-body energy in (a) PNO-LCCSD­(T) for acetylene, (b) DLPNO-CCSD­(T) for formic acid, (c) LNO-CCSD­(T) for ethylene, and (d) LNO-CCSD­(T) for ammonia. The data were calculated with AVTZ→AVQZ extrapolation.

The SCF convergence is not a factor in those issues because, in all cases, we use the extremely tight SCF convergence settings specified in the Technical Details section.^40^ Therefore, the divergences or shift can be attributed to the shortcomings of the off-the-shelf settings of the local approximations.

In the three-body term, divergences and drifts at long distances are more common because of the large number of trimers contributing up to a given distance cutoff. In fact, none of the off-the-shelf threshold settings shows qualitatively correct long-range distance behavior of the three-body nonadditive energy across the complete test set. Aiming at overcoming these difficulties, first, we identify some of the worst examples:

i Variants of PNO-LCCSD­(T) show drift in acetylene (Figure 2a), ethylene (Figure S5b), and divergence in ammonia (Figure S5c),
ii DLPNO-CCSD­(T)/Normal diverges in methanol (Figure 2b) and formic acid (Figure S7d),
iii all variants of LNO-CCSD­(T) show drift in ethylene (Figure 2c), while Normal and Tight settings diverge in ammonia (Figure 2d).

**Figure 2:**
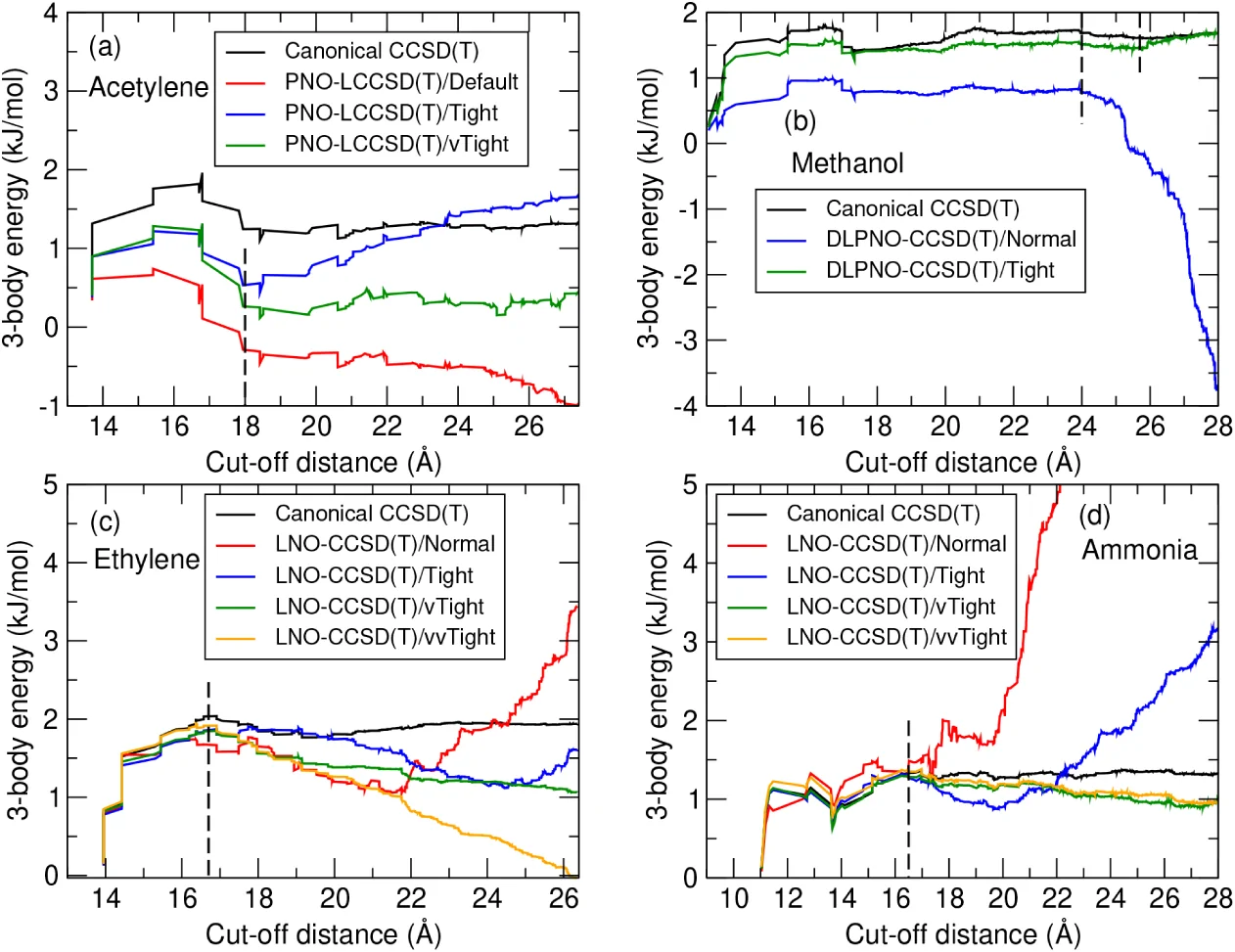
Distance convergence of the correlation contribution to the three-body nonadditive energy in (a) PNO-LCCSD­(T) for acetylene, (b) DLPNO-CCSD­(T) for methanol, (c) LNO-CCSD­(T) for ethylene, and (d) LNO-CCSD­(T) for ammonia. The data were calculated with AVTZ basis set. The DLPNO-CCSD­(T)/Loose results were omitted to avoid distorting the scale of the plot.

In Section 3.2 we analyze these difficulties and show how to overcome them. Besides the qualitative behavior, let us also note that when the three-body terms converge, the “normal/default” variants of the tested methods are often outside of the 1 kJ/mol precision mark, while tighter settings provide systematic improvement toward the canonical reference for both the two- and three-body terms.

#### 3.1.2 Basis-Set Convergence

There is a relatively fast basis-set convergence of the three-body correlation energy of the considered systems, at least for the wavefunction-based methods.^37,40^ Accordingly, the AVTZ basis set is used for the three-body CCSD­(T) calculations, and their basis-set convergence is not analyzed further. In contrast, two-body correlation energies converge slowly with basis set and often require CBS extrapolation.^37,40^ It is therefore of interest to examine how the separate CCSD and (T) correlation energy terms from localized coupled-cluster methods converge with basis-set size relative to their canonical counterparts.

To understand the differences for basis-set convergence we calculate the difference between values obtained with the AVQZ and AVTZ basis sets and show them in Table 1. Both LNO-CCSD and PNO-LCCSD provide similarly highly accurate description of the canonical CCSD basis-set convergence. Indeed, these methods with Tight or stricter thresholds, reproduce the AVQZ–AVTZ differences with at worst 0.15 kJ/mol error for all the considered systems. In comparison, DLPNO-CCSD with both Normal and Tight settings performs less consistently, overestimating the AVQZ–AVTZ difference by less than 0.2 kJ/mol for hydrocarbons, but by up to 0.3–0.6 kJ/mol for formic acid (Table 1).

**Table 1:** AVQZ–AVTZ Differences in 2-Body CCSD and (T) Energy Components (kJ/mol) from Canonical CCSD­(T), Together with Errors in These Differences from Local Coupled-Cluster Methods Relative to the Canonical Values

Threshold	Ethane	Ethylene	Acetylene	Ammonia	Methanol	Formic acid
**Canonical CCSD**						
–	–0.31	–0.53	–0.75	–0.77	–1.45	–2.45
PNO-LCCSD						
Tight	–0.11	–0.07	–0.11	0.00	–0.04	–0.11
vTight	–0.06	–0.05	–0.14	0.04	0.03	–0.06
DLPNO-CCSD						
Normal	–0.09	–0.22	–0.12	–0.11	–0.15	–0.57
Tight	–0.14	–0.05	–0.13	–0.04	–0.03	–0.34
LNO-CCSD						
Normal	–0.02	–0.16	–0.01	0.04	–0.02	0.55
Tight	0.00	0.07	–0.01	–0.11	0.09	0.07
vTight	0.01	–0.02	0.04	0.03	0.08	0.00
vvTight	–0.01	0.00	–0.15	0.04	0.01	–0.03
**Canonical (T)**						
–	–0.17	–0.19	–0.20	–0.19	–0.29	–0.36
PNO-L(T)						
Tight	0.13	0.16	0.13	0.13	0.22	0.35
vTight	0.12	0.15	0.06	0.10	0.18	0.20
DLPNO-(T)						
Normal	0.11	0.09	0.11	0.13	0.16	0.02
Tight	–0.10	0.05	0.07	0.08	0.12	0.12
LNO-(T)						
Normal	0.90	0.36	–0.21	–0.17	0.48	0.09
Tight	0.00	–0.07	0.08	0.08	0.12	0.04
vTight	0.06	0.01	–0.04	0.03	0.10	0.07
vvTight	0.02	0.01	0.00	0.06	0.03	0.04

The canonical (T) contribution exhibits much smaller AVQZ–AVTZ differences than the CCSD term, ranging from −0.17 to −0.36 kJ/mol for the studied systems, see Table 1. Within this narrow range of basis-set differences, especially from the perspective of our strict absolute and relative precision targets, LNO-(T) matches the canonical trends best. Indeed, when the Tight threshold is applied, the LNO-(T) AVQZ–AVTZ increments remain within 0.1 kJ/mol of the canonical values, and the use of vTight or vvTight thresholds further reduces these discrepancies (Table 1). In comparison, PNO-L­(T) and DLPNO-(T) show larger deviations from the canonical reference, reaching 0.4 kJ/mol for PNO-L­(T)/Tight in the case of formic acid (Table 1). Considering the combined CCSD­(T) values, LNO-CCSD­(T) is found to provide a consistently accurate description also for the basis-set convergence. The PNO- and DLPNO-based approaches exhibit considerable deviations from the canonical CCSD­(T) reference, primarily because of their (T) contributions. A similar trend has been reported before when comparing the (T) contributions of LNO-(T) to PNO-based (T) corrections in cases of large (T) contributions.^43,82^ This can be related to the different (T) approximation methodology used for LNO-(T) compared to the PNO-based methods.^43,47^ For completeness, one may consider alternative basis set convergence accelerator methods besides CBS extrapolation,^83–85^ such as explicitly correlated or density based basis set correction^86^ based variants of these local correlation methods. As long as the local approximations perform similarly well for the triple- and quadruple-*ζ* basis sets, CBS extrapolation is equally suitable.

#### 3.1.3 Accuracy and Computational Cost

Here we discuss the accuracy of the local CCSD­(T) binding energy of the considered systems compared to the canonical reference, as shown in Figure 3. The two-body CCSD, (T), and total CCSD­(T) absolute binding energies obtained from the examined methods for all systems are provided in Tables S7–S9 of Supporting Information.

**Figure 3:**
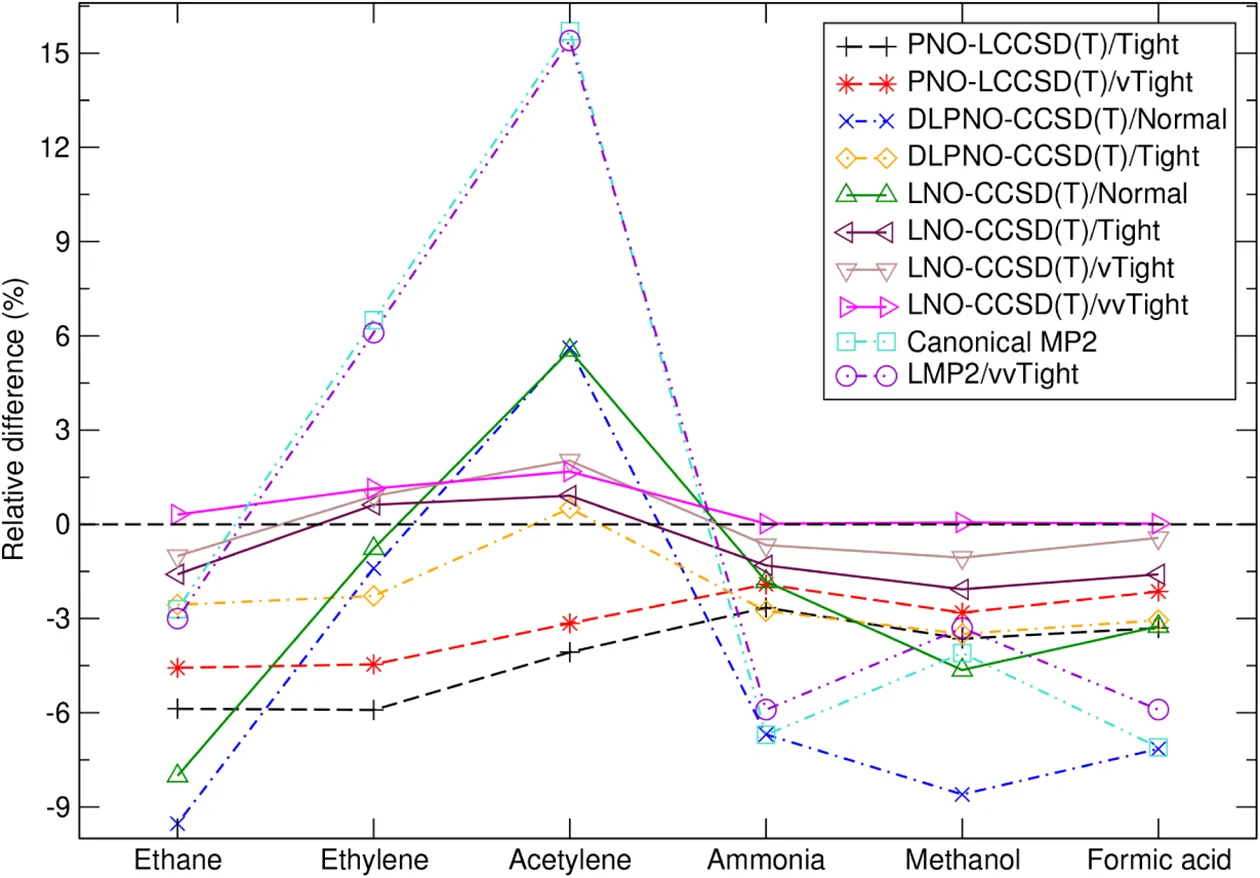
Relative differences between approximate two-body energies and the canonical CCSD­(T) reference. PNO-LCCSD­(T)/Default and DLPNO-CCSD­(T)/Loose are omitted due to large errors.

For all local approximations, the errors in the two-body interaction energies relative to the canonical CCSD­(T) reference are systematically reduced upon incrementing the threshold set for most of systems (Figure 3). For PNO-LCCSD­(T)/Tight, the two-body energies are too weak by around 3–6%. The stricter vTight settings bring an error reduction by about one per cent. DLPNO-CCSD­(T)/Normal yields a large spread of errors, from nearly −10% for ethane to almost 6% for acetylene. DLPNO-CCSD­(T)/Tight reduces the spread to between −4% and 1%. Notably, the main source of the errors in both PNO-L and DLPNO approaches is the (T) component of the interaction energy. Indeed, for the CCSD component, the PNO-L/vTight and DLPNO/Tight schemes give errors below 6%, while the (T) contributions are too large by 15–40% (Figures S2–S3 in Supporting Information).

LNO-CCSD­(T)/Normal yields relative errors between −8 and 6 percent, comparable to DLPNO-CCSD­(T)/Normal. LNO-CCSD­(T)/Tight reduces the errors to between −2 and 1%, consistently outperforming both PNO-LCCSD­(T)/vTight and DLPNO-CCSD­(T)/Tight. The LNO-CCSD­(T)/vvTight results are essentially error-free for ethane and hydrogen-bonded systems. Residual deviations of 1–2% remain for systems with delocalized electrons. The LMP2/vvTight method, added as an additional test of the LNO family of approximations, closely replicates the canonical MP2 results.

Overall, LNO-CCSD­(T) with sufficiently tight thresholds is found to be the most reliable method examined. However, one needs to consider also the computational cost of the different approximations. To this end, we collect the computational times for the calculation of two-body contributions of methanol (Figure S17 of Supporting Information). Clearly, the computational cost of LNO-CCSD­(T) increases with tightening the thresholds. Here we find about 2.5 times higher cost when switching from Tight to vTight or from vTight to vvTight. The vvTight settings then give a speed-up of around four compared to the canonical calculations. The computational cost of the PNO-LCCSD­(T)/vTight and DLPNO-CCSD­(T)/Tight calculations is between that of LNO-CCSD­(T) with vTight and vvTight settings for methanol dimers. We note that the speedups should be markedly higher for monomers larger than methanol.

We note that errors in the two-body contributions of approximate methods relative to the reference CCSD­(T) results occur mainly at short intermolecular distances.^37,40^ This behavior raises the question of whether LNO-CCSD­(T)/vvTight thresholds are necessary at large distances, or whether looser LNO-CCSD­(T) settings can be used instead while still reproducing vvTight-level results across the entire distance range. To address this, we evaluated the accuracy of a mixed-threshold protocol in which LNO-CCSD­(T)/vvTight was applied at short distances (<10 Å) and LNO-CCSD­(T)/Normal at larger distances (>10 Å) for the two-body contributions of all the considered systems. The results show that this approach reproduces LNO-CCSD­(T)/vvTight two-body contributions over the entire distances with negligible errors, below 0.02 kJ/mol for most systems and at most 0.15 kJ/mol for ammonia and formic acid. Thus, this scheme can be used as an efficient strategy to reduce computational cost in local coupled-cluster calculations for two-body contributions.

A direct comparison of the three-body CCSD­(T) correlation energies obtained from localized-orbital coupled-cluster approaches with the reference canonical CCSD­(T) results is not reliable over the entire distance range considered, owing to numerical noise at large separations (Figure 4). Nevertheless, at short distances, LNO-CCSD­(T)/vvTight generally yields higher accuracy than PNO-LCCSD­(T)/vTight or DLPNO-CCSD­(T)/Tight. In the following section we identify LNO-CCSD­(T) settings that improve the distance convergence of the three-body LNO-CCSD­(T) contributions at large distances.

**Figure 4:**
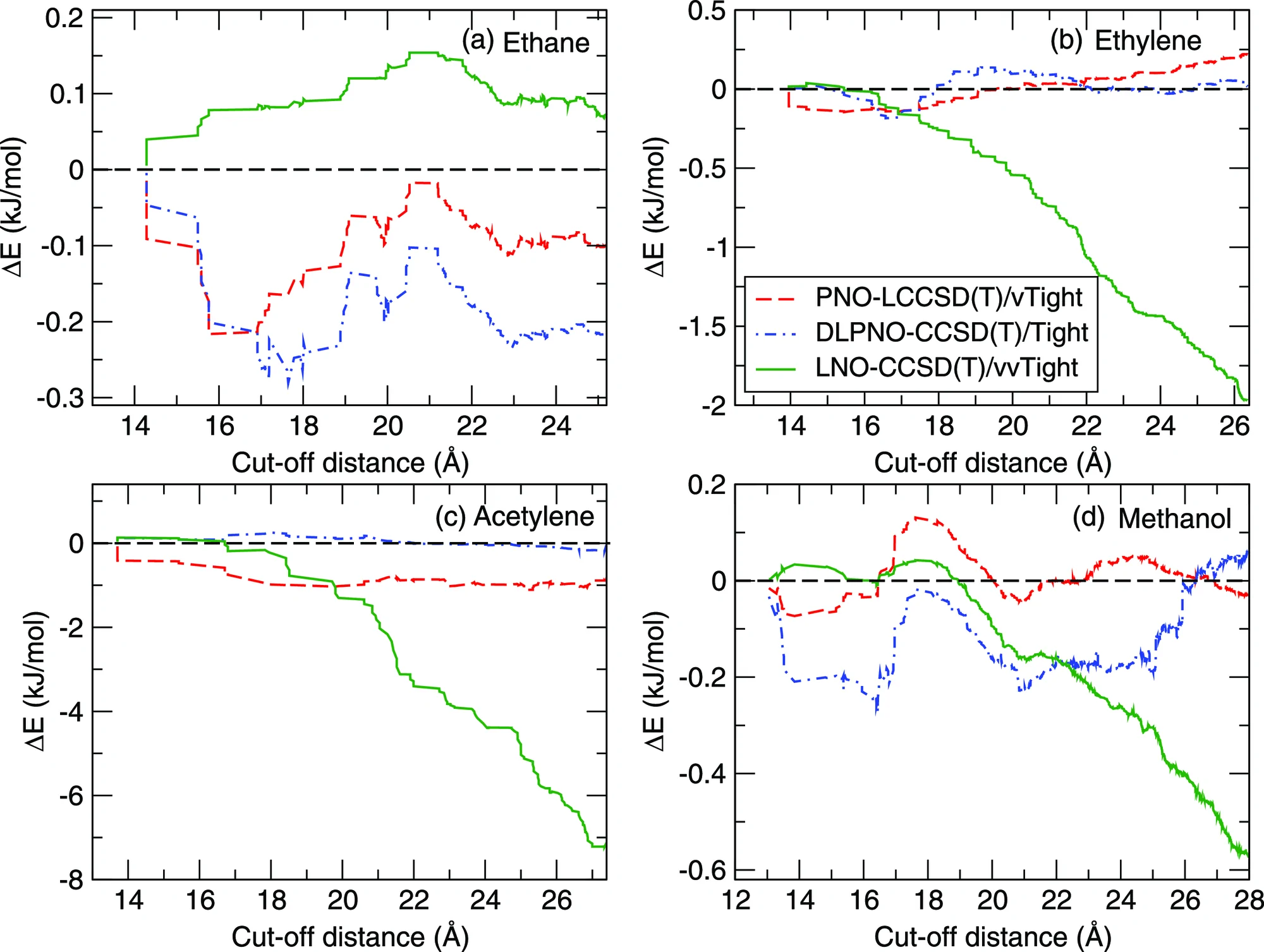
Differences between approximate three-body energies and the canonical CCSD­(T) reference as a function of distance for (a) ethane, (b) ethylene, (c) acetylene, and (d) methanol.

### 3.2 Improved LNO-CCSD­(T) for Many-Body Interactions

#### 3.2.1 New Thresholds

To identify and resolve the origin of the distance-convergence issues in the three-body LNO-CCSD­(T) correlation energy at large intermolecular separations, we separately and systematically analyzed the effect of individual approximations made within the LNO approach. The long-range behavior was not in the focus of a previous study by some of us, as vvTight LNO-CCSD­(T) was employed there for the two-body term up to 7 Å.^15^ In parallel, compared to the localcc=2021 version of LNO-CCSD(T) employed in ref 15, improvements have been made to the localcc=2024 version. For example, it has a more advanced atomic and virtual orbital definition,^43,48^ particularly useful for the counterpoise approach. Moreover, this virtual orbital domain and another (i.e., MP2 denominator factorization) approximation were tightened for localcc=2024, because they also helped to neutralize the accumulation of three-body noise, as shown for ice crystals against canonical CCSD­(T).^31^ This LNO-CCSD­(T) version was used to obtain short-range two- and three-body terms and combined with the D4 dispersion model for the mid–long-range two- and three-body terms in ref 31, thus the use of LNO-CCSD­(T) for the long-range contributions has not yet been thoroughly investigated.

We aim at benchmark precision, ca. 0.1 kJ/mol, compared to canonical CCSD­(T), even when using LNO-CCSD­(T) for very long-range three-body contributions. Starting from the default localcc=2024 version of LNO-CCSD­(T), we found that high precision can be achieved by tightening the threshold of some of the LNO approximations (see new values in Table 2), while also optimizing for moderate increase in the computational cost. The steepest sensitivity in computational cost corresponds to the occupied and virtual natural orbital occupation number thresholds, governed by the lnoepso and lnoepsv keywords, respectively. The next tier of cost increase would originate from the main thresholds governing the strongly interacting local orbital pair list (wpairtol) as well as the atomic orbital (AO) and projected atomic orbital (PAO) bases in the domain approximation (bpedo) of the LNO approach. Fortunately, these approximations can remain in their previous settings even when aiming for a few tenths of a kJ/mol LNO errors.

**Table 2:** Modified Precision Thresholds for the LNO Coupled-Cluster Method in Mrcc ^a^

Threshold	Normal	Tight	vTight	vvTight	vvTight+
bpedo	0.9999	0.99995	0.99999	0.999995	0.999995
wpairtol [E_h_]	1 × 10^–5^	3 × 10^–6^	1 × 10^–6^	3 × 10^–7^	**3 × 10^–8^**
lnoepso	1 × 10^–5^	3 × 10^–6^	1 × 10^–6^	3 × 10^–7^	3 × 10^–7^
lnoepsv	1 × 10^–6^	3 × 10^–7^	1 × 10^–7^	3 × 10^–8^	3 × 10^–8^
naf_cor [E_h_]	1 × 10^–2^	**3** **× 10^–3^**	1 × 10^–3^	**3** **× 10^–4^**	**3** **× 10^–4^**
iesttol	**0.1**	**0.05**	**0.01**	**0.005**	**0.005**

a Bold values indicate changes of the LNO thresholds compared to the localcc = 2024 values, which are listed in Table S3 of Supporting Information. In addition, we use an additional precision level, vvTight+. Note that we also set scfdtol = 8 and cctol = 6 in our calculations.

To simplify the analysis, we only present in detail the investigation of the individual approximations starting from using the vvTight threshold set for the unchanged variables. First, a tighter SCF convergence threshold than the current default (scfdtol = 7) is set, motivated by previous works on accurately describing long-range interactions and reducing numerical errors in *n*-body contributions in the binding energies of molecular solids.^31,40,66^ Specifically, our test indicates that scfdtol = 8 is sufficient to achieve converged results (see Figure S10 in Supporting Information), and we used this value henceforth. In addition, we find that the convergence threshold for the CCSD iteration benefits from tightening its default value (cctol = 5), in agreement with ref 31. Indeed, as shown in Figure 5a, using cctol = 6 significantly improves the distance-convergence behavior of the three-body CCSD­(T) correlation energy of acetylene.

**Figure 5:**
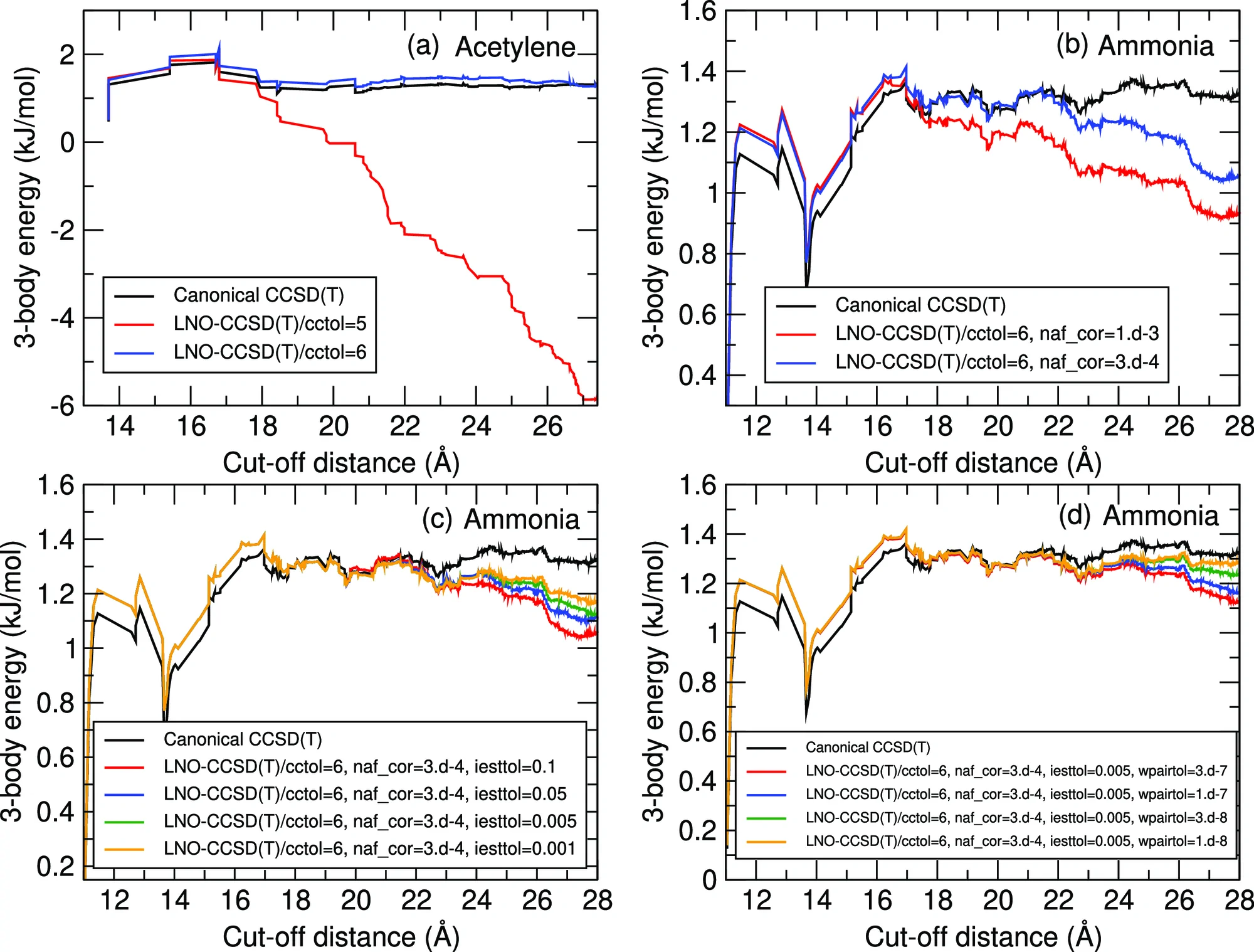
Distance convergence of the correlation contribution to the three-body nonadditive energy in LNO-CCSD­(T) using different settings of (a) cctol for acetylene, (b) naf_cor for ammonia, (c) iesttol for ammonia, and (d) wpairtol for ammonia.

Turning to the remaining LNO approximations, natural auxiliary functions (NAFs), governed by the NAF occupation number threshold naf_cor, compress the auxiliary basis required for the density-fitting approximation.^48^ As shown in Figure 5b, tightening the NAF approximation beyond its default value naf_cor = 10^–3^ in the previous vvTight settings pushes the distance cutoff range with drift-free convergence from 16 to 22 Å. Further tightening of naf_cor does not yield additional improvement.

At this point, the combination of the tightened scfdtol, cctol and naf_cor settings could be recommended for robust sub-kJ/mol performance in the three-body LNO-CCSD­(T) contributions. This holds especially in practical use when one can stop at a cutoff distance indicating convergence or when the very long-range three-body contributions can be estimated with lower-cost models instead of LNO-CCSD­(T) (see Section “Three-Body Terms”). It is noteworthy that all three adjustments in scfdtol, cctol, and naf_cor should bring only minor increase in the computational cost on the magnitude of ten percent.

Next, we continue the analysis motivated by the goal of using LNO-CCSD­(T) for very long-range three-body interactions aiming at benchmark level convergence on the scale of 0.1–0.2 kJ/mol. To that end, the next level of improvement is provided by tightening the iesttol threshold,^48,49^ which governs the selection of additional AOs and PAOs to the domain approximation (see Figure 5c). Such an extension is usually found impactful in the context of the counterpoise approach, when there are very low localized-orbital coefficients on the ghost AO, but these ghost AOs still contribute considerably to the AO basis set expansion.^48^ Improving iesttol can cut in half the remaining LNO error in the long-range three-body contribution (Figure 5c). The remaining LNO error can be diminished for ammonia (Figure 5d) via extending the strong local molecular orbital pair list of the LNO methods, governed by the wpairtol keyword. Due to the cost increase associated with tightening wpairtol, we put together a new tier of vvTight+ settings including this improvement, that is recommended for benchmark precision three-body energy contributions up to very long-range distance cutoffs.

Based on the presented and all other tests, we propose LNO-CCSD­(T) settings summarized in Table 2. In the following, we reassess the accuracy of LNO-CCSD­(T) with these new settings for both two-body and three-body contributions to the binding energy of the considered systems.

#### 3.2.2 Two-Body Terms

We first examine the effect of the new LNO settings on the two-body correlation energies and assess the extent to which they improve agreement with the reference values. The differences in LNO-CCSD­(T) correlation energy between the localcc=2024 version and new settings for all the considered systems are summarized in Table S10 of Supporting Information, with ammonia shown in Figure 6 as one of the most difficult examples from Section “New Thresholds”.

**Figure 6:**
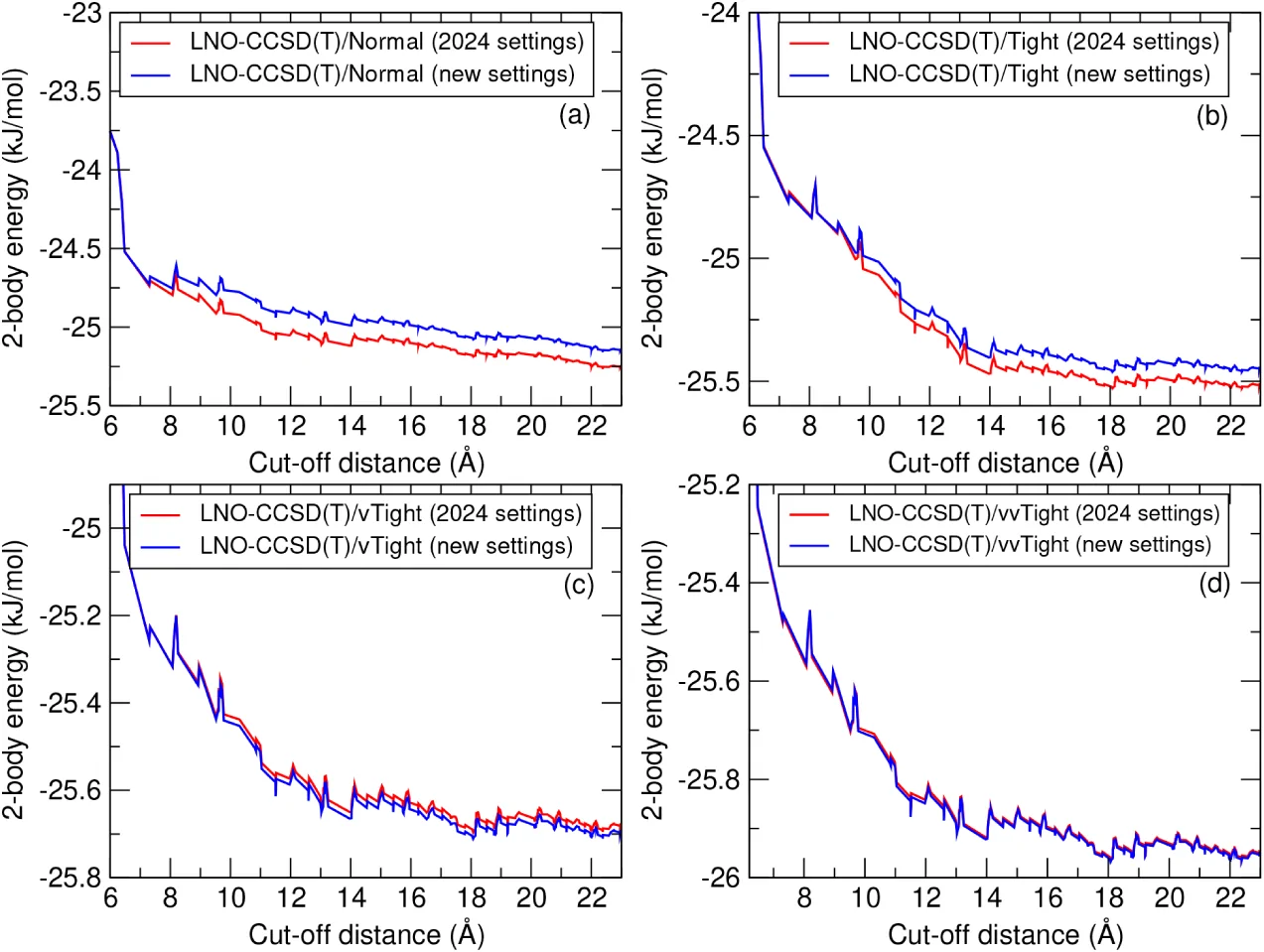
Distance convergence of the correlation contribution to the two-body energy of ammonia in (a) LNO-CCSD­(T)/Normal, (b) LNO-CCSD­(T)/Tight, (c) LNO-CCSD­(T)/vTight, and (d) LNO-CCSD­(T)/vvTight using 2024 (localcc=2024) and new settings. The data were calculated with AVTZ → AVQZ extrapolation.

The performance of the LNO-CCSD­(T) method with the new settings is very similar to that of the 2024 settings for the two-body terms, see Figure 6. The differences in LNO-CCSD­(T) correlation energy between the two settings are generally below 0.1 kJ/mol. However, an exception is observed for the Normal threshold in methanol and formic acid, where the differences reach 0.2–0.3 kJ/mol, see Table S10 in Supporting Information.

Next, we analyzed how the series of Normal, Tight, vTight, etc. LNO-CCSD­(T) results can be employed for accelerating the convergence toward canonical CCSD­(T) as well as for the estimation of the remaining LNO uncertainties. The foundation of this is that the approximations in LNO-based approach are controlled by a hierarchy of increasingly tighter settings and typically lead to systematic improvement toward the canonical (local-approximation-free, LAF) limit.^43,49^ Although strict monotonic convergence is not guaranteed in all cases due to bundling multiple individually converging approximations, in practice the energies generally improve systematically as the thresholds are tightened.^43,49^ This behavior enables a simple extrapolation toward the LAF limit using two consecutive settings *S* and *S*  + 1,

6 $$E_{\mathrm{LAF}}^{S-(S+1)}=E_{S+1}+\frac{1}{2}\left(E_{S+1}-E_{S}\right)$$

where *S* denotes the LNO setting series (Normal, Tight, vTight, etc.), yielding the corresponding Normal–Tight, Tight–vTight, etc., extrapolated values.^43,49^

We now examine the accuracy of the extrapolation to the LAF limit for the two-body correlation contribution using data obtained with the new LNO settings. As shown in Figure 7, the Normal–Tight extrapolation greatly outperforms Normal, is better than Tight for hydrogen-bonded systems, and yields errors within 2% of the reference. Similarly Tight–vTight extrapolation improves on both Tight and vTight in terms of the parallelity of the error curve as well as reducing the relative errors to below 1% for all systems except acetylene (see Figure 7). These results indicate that Normal–Tight can be used instead of vTight and the Tight–vTight extrapolation yields results close to canonical CCSD­(T), so these LAF extrapolations can be recommended for larger systems when vTight or vvTight LNO-CCSD­(T) would be too computationally demanding. Finally, a comparison of the computational time obtained with the 2024 settings and the new settings is presented in Figure S18 of Supporting Information, showing that the new settings lead to a slight increase in timings.

**Figure 7:**
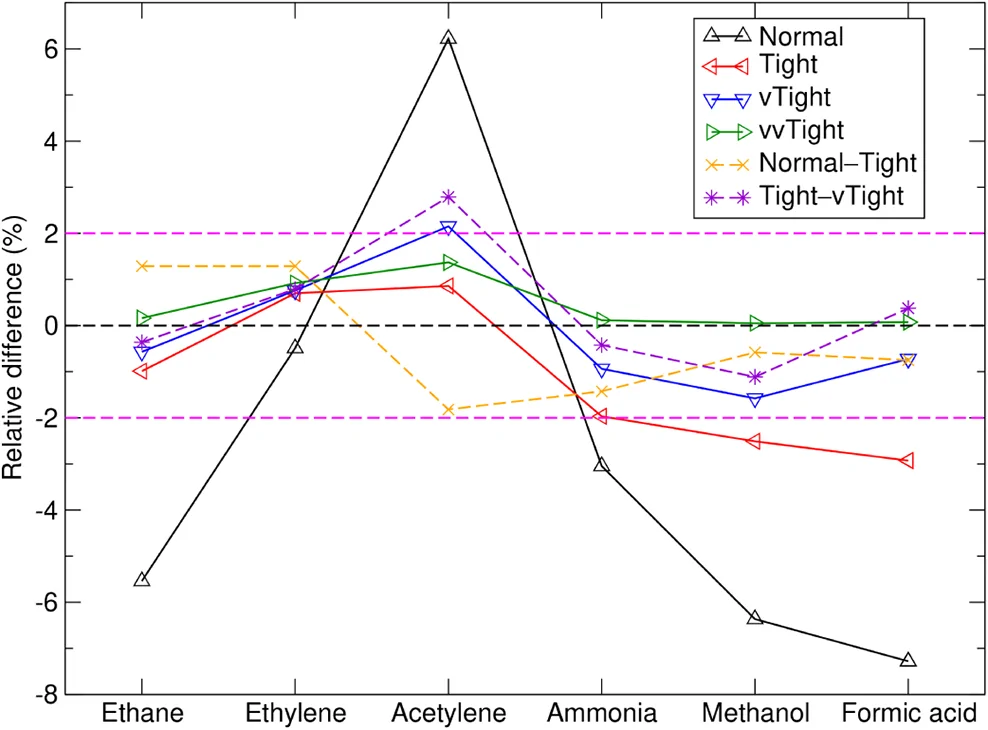
Performance of the LNO-CCSD­(T) method for the two-body correlation contribution using extrapolation approach. The updated LNO thresholds from Table 2 were used.

#### 3.2.3 Three-Body Terms

Let us examine the performance of the final, updated LNO-CCSD­(T) precision tiers in the three-body energy contributions. Acetylene and methanol are highlighted as stark examples of the numerical artifacts at long distances (Figure 8). The results for the remaining systems are available in Figures S11–S12 in Supporting Information. The benchmark tier, LNO-CCSD­(T)/vvTight+, shows no numerical artifacts at long distances for any of the six tested systems. The deviations with respect to the canonical CCSD­(T) reference are all within 0.2 kJ/mol. The vvTight tier clearly benefits from the updated thresholds. The divergence is eliminated in all cases except methanol, where the error drifts to 0.6 kJ/mol at the far end of the inspected range. In systems other than methanol, updated LNO-CCSD­(T)/vvTight is excellent and matches the reference to within 0.2 kJ/mol.

**Figure 8:**
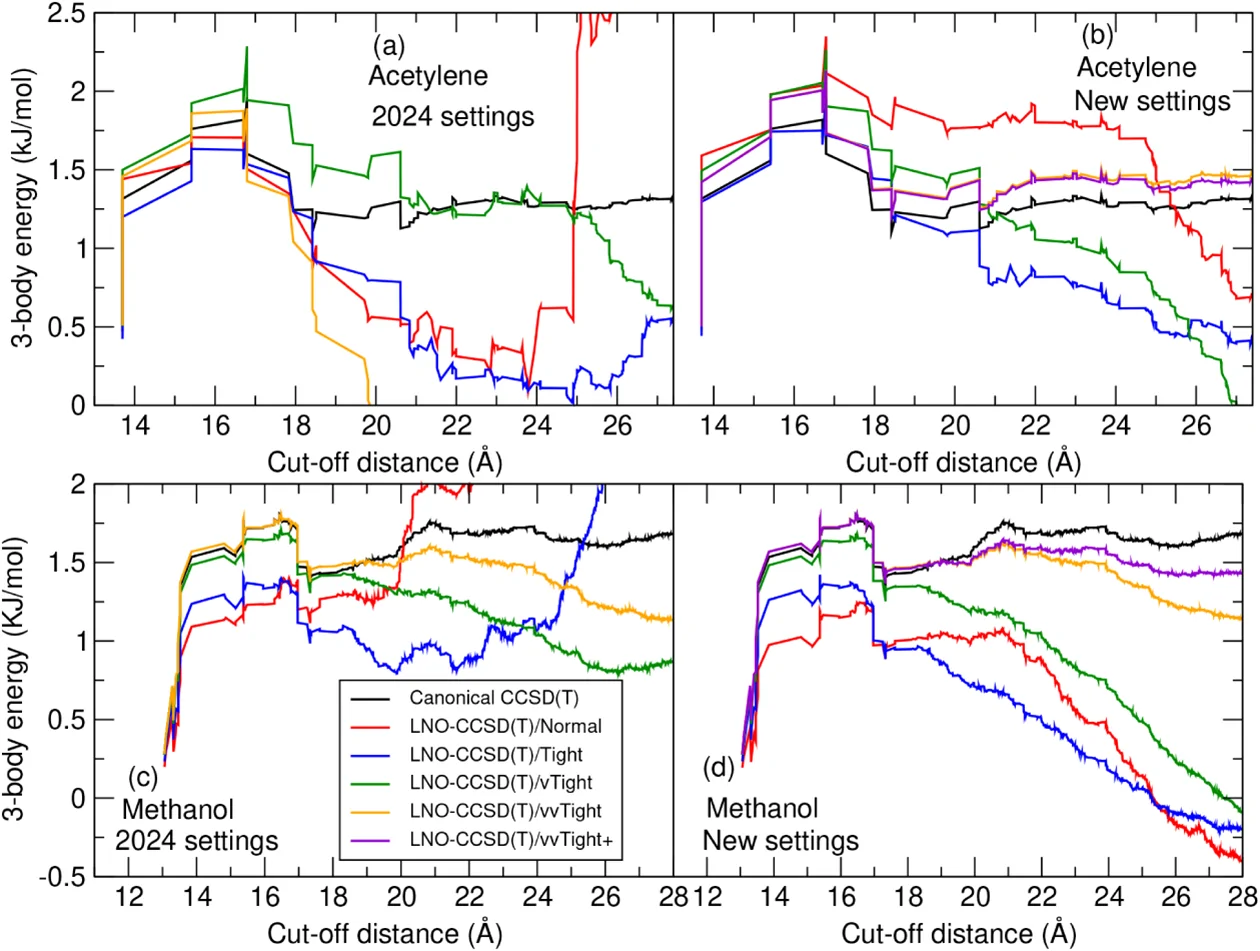
Distance convergence of the LNO-CCSD­(T) correlation contribution to the three-body energy: (a) acetylene with the 2024 settings, (b) acetylene with the new settings, (c) methanol with the 2024 settings, and (d) methanol with the new settings.

For the routine levels of LNO-CCSD­(T), i.e., Normal, Tight, and vTight, the divergence at long distances persists for acetylene, methanol, ammonia, and formic acid. However, despite the challenges at long distances, the nonadditive energies of compact trimers are all well-behaved and show systematic improvement across all tiers of predefined LNO settings (Figure 8). Specifically, at a cutoff of *R* = 17 Å, *i.e.*, before the onset of the drift, the maximum errors of the LNO-CCSD­(T) methods are 0.2 kJ/mol (vvTight), 0.3 kJ/mol (vTight), 0.7 kJ/mol (Tight), and 1.2 kJ/mol (Normal), see Figure S15 in Supporting Information.

Similar to the behavior observed for LNO-CCSD­(T), LMP2 with thresholds looser than vvTight+ exhibits numerical divergence or drift in the three-body interaction energies at very large separations (see Figure S14). The LMP2/vvTight+ three-body contributions are in excellent agreement with the canonical MP2 results (Figure 10).

Our observations suggest a pragmatic multi-level strategy, in which LNO-CCSD­(T) is applied only to short-range trimers and the long-range trimers are accounted for using a numerically robust implementation of a lower-level theory. A suitable candidate for the lower-level method is RPA or one of its extensions, as they describe the three-body Axilrod–Teller–Muto correlations. Here, we choose the RPA+ph method,^15^ which is appealing due to its properly antisymmetrized particle-hole terms in the third order of perturbation theory.

As illustrated in Figures 9 and S13, the multi-level approach succeeds in removing the artifacts in the long-range part of the three-body curve. In the most challenging case where the three body artifacts appear in pure LNO-CCSD­(T), methanol, the energy error is practically independent of distance anywhere in the RPA+ph region (Figure 9). This is true for the entire dataset, with the additional examples of ethane, acetylene, and formic acid shown in Figures S13 and S16 in Supporting Information. The overall accuracy of the multi-level three-body contribution is controlled by the LNO settings at short range. For the switchover radius of *R* = 17 Å, the maximum errors depending on the LNO precision are as follows: 0.3 kJ/mol for vvTight, 0.4 kJ/mol for vTight, 0.8 kJ/mol for Tight, and 1.2 kJ/mol for the least accurate Normal setting (Figure 10). In all cases where the multi-level approach deviates from the reference by more than 0.2 kJ/mol, the error can be traced back to the compact trimers in the LNO-CCSD­(T) part, cf. Figure S15 in Supporting Information.

**Figure 9:**
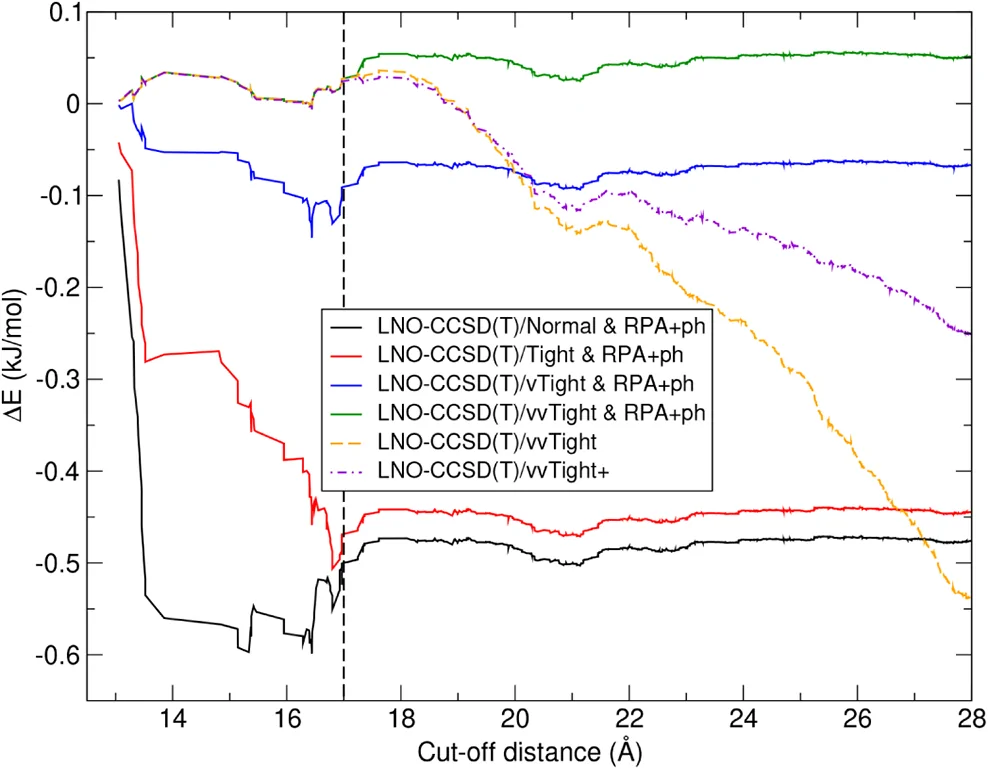
Distance-dependent differences between the approximate and reference nonadditive three-body energies of methanol.

**Figure 10:**
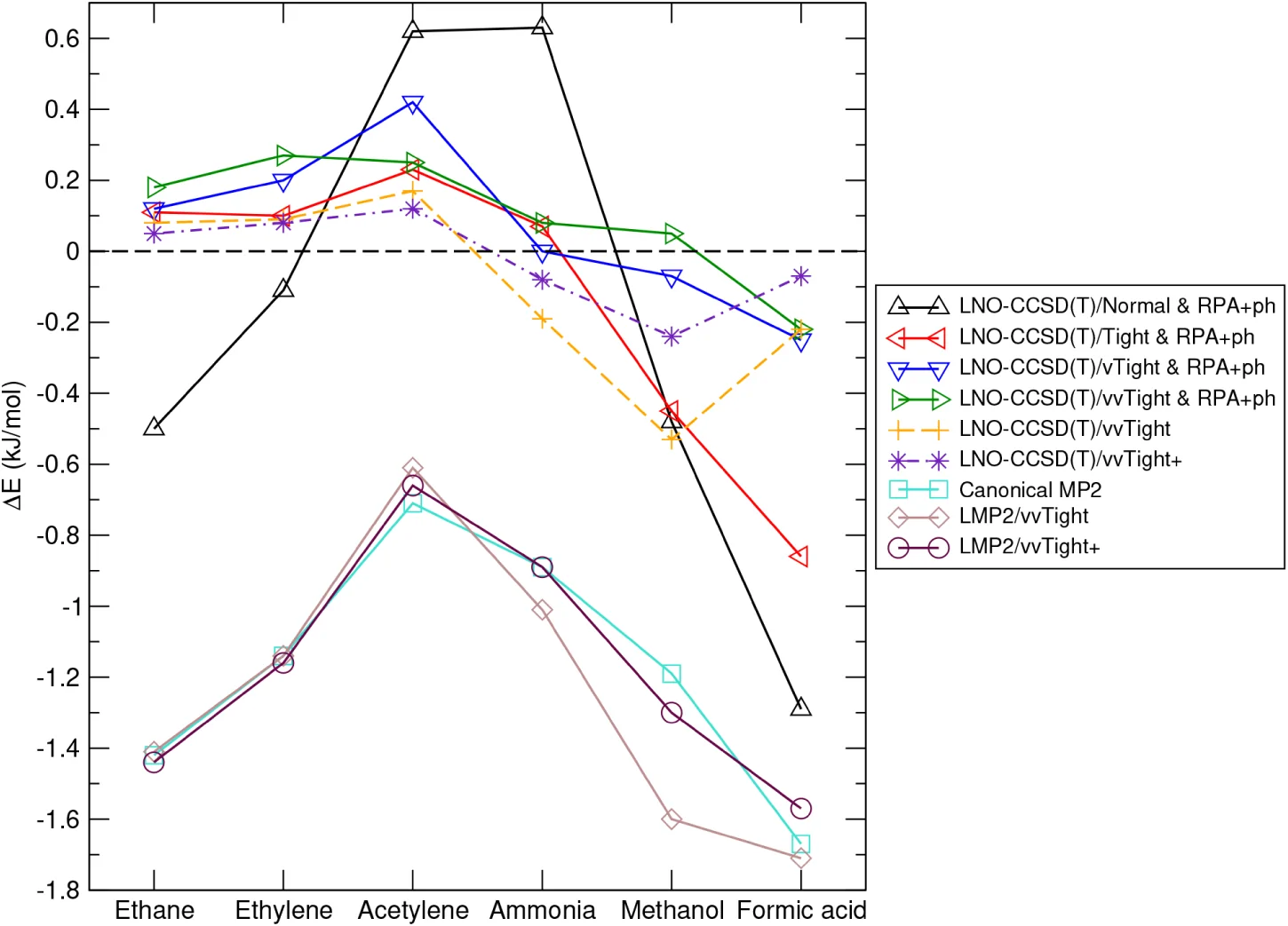
Differences in the three-body energies predicted by the LNO and multi-level methods relative to the canonical CCSD­(T) reference.

#### 3.2.4 Total Lattice Energies

Our findings on the individual two- and three-body contributions suggest the following well-balanced computational strategies for the total lattice energy. For benchmark accuracy, one should employ LNO-CCSD­(T)/vvTight for dimers and a combination of LNO-CCSD­(T)/vvTight and RPA+ph for trimers. Routine calculations should utilize the LAF extrapolation in dimers and a combination of LNO-CCSD­(T) and RPA+ph in the trimer contributions. The strategies, referred to as multi-level­(*X*), where *X* is the precision of the LNO-CCSD­(T) component, are listed in Table 3.

**Table 3:** LNO-CCSD­(T) Method and Multi-Level Approaches Based on a Combination of LNO-CCSD­(T) and RPA+ph

Label	Two-body terms^a^	Three-body terms^b^	Embedding^c^	Avg error^d^	Max error^d^
LNO-CCSD(T)/vvTight+	vvTight^e^	vvTight+	HF(PBC)	0.14	0.25
multi-level(Tight)	LAF Normal-Tight	Tight & RPA+ph	HF(PBC)	0.42	0.66
multi-level(vTight)	LAF Tight-vTight	vTight & RPA+ph	HF(PBC)	0.24	0.40
multi-level(vvTight)	vvTight	vvTight & RPA+ph	HF(PBC)	0.09	0.24

a LNO-CCSD­(T) is applied to all dimers.

b Multi-level approach: LNO-CCSD­(T) below the switchover distance, RPA+ph for the rest.

c Periodic HF correction accounts for clusters outside of the cutoff radii and higher many-body energy contributions.

d Average and maximum errors in total lattice energies in kJ/mol.

e The vvTight and vvTight+ settings yield nearly identical dimer energies.

The total lattice energies, obtained by combining the two-body, three-body, and beyond-three-body contributions for different computational strategies, are summarized in Table 4 and the average and maximum errors on our test set are given in Table 3. The multi-level­(vvTight) approach achieves the maximum error of 0.2 kJ/mol in the total lattice energies in our test set. Almost equivalent results are obtained if LNO-CCSD­(T)/vvTight+ is used for the entire range of trimers. At this point, the error due to the local approximation in coupled clusters and the use of a lower level of theory in long-range trimers is most probably below the background of errors from other sources, such as the incomplete convergence of MBE-resolved correlation contributions with distance. The multi-level­(vTight) approach is only slightly less accurate than the benchmark, with the maximum error of 0.4 kJ/mol for the systems tested. The efficient multi-level­(Tight) strategy affords the maximum numerical errors of ca. 0.7 kJ/mol, see Figure 11. Therefore, the multi-level protocols defined in Table 3 form a series of approximations which systematically converge to the benchmark-level precision of the total lattice energy.

**Table 4:** Two-Body, Three-Body, and Beyond-Three-Body Contributions (Δ*E* ^HF^(PBC)), Together with the Total Binding Energies

Systems	Methods	Two-body	Three-body	Δ*E* ^HF^(PBC)^a^	Total
Ethane	Canonical CCSD(T)	–23.86	1.46	0.01	–22.39
	LNO-CCSD(T)/vvTight+	–23.92	1.51		–22.40
	multi-level(Tight)	–24.33	1.57		–22.75
	multi-level(vTight)	–23.73	1.58		–22.14
	multi-level(vvTight)	–23.92	1.64		–22.27
Ethylene	Canonical CCSD(T)	–23.53	1.56	–0.11	–22.08
	LNO-CCSD(T)/vvTight+	–23.82	1.64		–22.29
	multi-level(Tight)	–23.96	1.66		–22.41
	multi-level(vTight)	–23.79	1.76		–22.14
	multi-level(vvTight)	–23.82	1.83		–22.10
Acetylene^b^	Canonical CCSD(T)	–25.95	1.15	–0.15	–24.95
	LNO-CCSD(T)/vvTight+	–26.26	1.27		–25.14
	multi-level(Tight)	–25.51	1.38		–24.28
	multi-level(vTight)	–26.60	1.57		–25.18
	multi-level(vvTight)	–26.26	1.40		–25.01
Ammonia^c^	Canonical CCSD(T)	–36.61	0.65	0.26	–35.70
	LNO-CCSD(T)/vvTight+	–36.64	0.57		–35.81
	multi-level(Tight)	–36.24	0.72		–35.26
	multi-level(vTight)	–36.50	0.65		–35.59
	multi-level(vvTight)	–36.64	0.73		–35.65
Methanol	Canonical CCSD(T)	–49.16	–5.93	0.01	–55.08
	LNO-CCSD(T)/vvTight+	–49.18	–6.17		–55.34
	multi-level(Tight)	–48.94	–6.38		–55.31
	multi-level(vTight)	–48.73	–6.00		–54.72
	multi-level(vvTight)	–49.18	–5.88		–55.05
Formic acid	Canonical CCSD(T)	–80.94	–1.13	–2.89	–84.96
	LNO-CCSD(T)/vvTight+	–80.97	–1.20		–85.06
	multi-level(Tight)	–80.64	–1.99		–85.52
	multi-level(vTight)	–81.09	–1.38		–85.36
	multi-level(vvTight)	–80.97	–1.35		–85.21

a Periodic mean-field embedding contribution is identical for all methods.

b Also referred to as acetylene form I (see Table S12).

c The CCSD­(T) reference lattice energy differs by 2.5 kJ/mol from the known diffusion Monte Carlo reference lattice energy,^14^ $E_{\mathrm{latt}}^{\mathrm{DMC}}=-38.2\pm 0.1$ kJ/mol, primarily due to the different geometries used.

**Figure 11:**
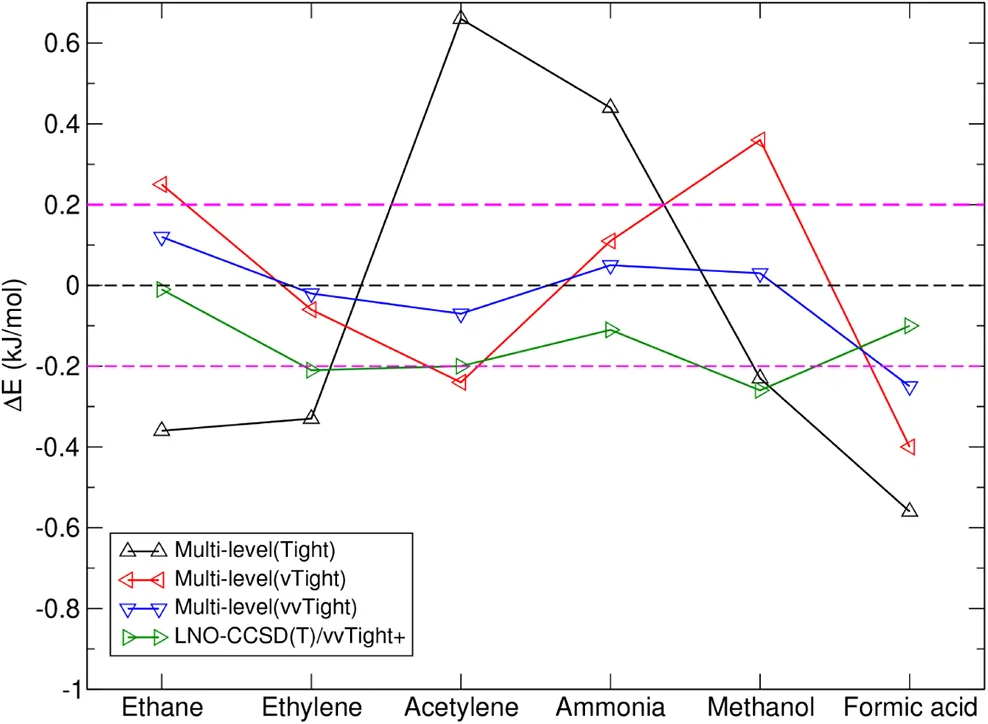
Differences between approximations of the total lattice energy and the canonical CCSD­(T) reference. The lattice energy contains two- and three-body contributions.

An important application of accurate theoretical methods is the energetic ranking of molecular crystal polymorphs, where relative lattice energies often differ by only a few kJ/mol.^62^ As a test case, we consider the differences between two polymorphs of acetylene: one in space group *Pa*3̅ (form I) and the other in space group *Cmce* (form II).^87^ The distance convergence of the two-body and three-body contributions is shown in Figures S19 and S20, and the corresponding binding energies are listed in Table S12 of Supporting Information. The distance convergence is nearly identical for the two polymorphs, indicating that the choice of cutoff distance and LNO precision settings affects both structures similarly (see Figures S19 and S20). All three multi-level approaches correctly predict form II to be more stable than form I, consistent with the canonical CCSD­(T) reference (Table S12).

### 3.3 Benchmarking Dispersion-Corrected DFT

It is interesting to test how useful are the LNO-based approaches as benchmarks for methods such as DFT. To this end, we select PBE-D4 and PBE0-D4 ^88–90^ and calculate the corresponding total binding energies within PBC using VASP and the two- and three-body energies (Table S13 of Supporting Information). The total errors for PBE-D4 and PBE0-D4 are rather small, they reach only a few kJ/mol or up to 10% (Figure 12). Such accuracy is sufficient to obtain a qualitative agreement with binding energies derived from experiment. However, the deviations of the two- and three-body energies are large, e.g., over 8 kJ/mol for ammonia (Figure 12). It is thus clear that the good results obtained for the total binding energy are due to fortuitous error cancellation between the different *n*-body errors. We note that the four- and five-body errors are non-negligible for PBE and PBE0 and cause the apparent disagreement between the total error and the sum of the two- and three-body errors.

**Figure 12:**
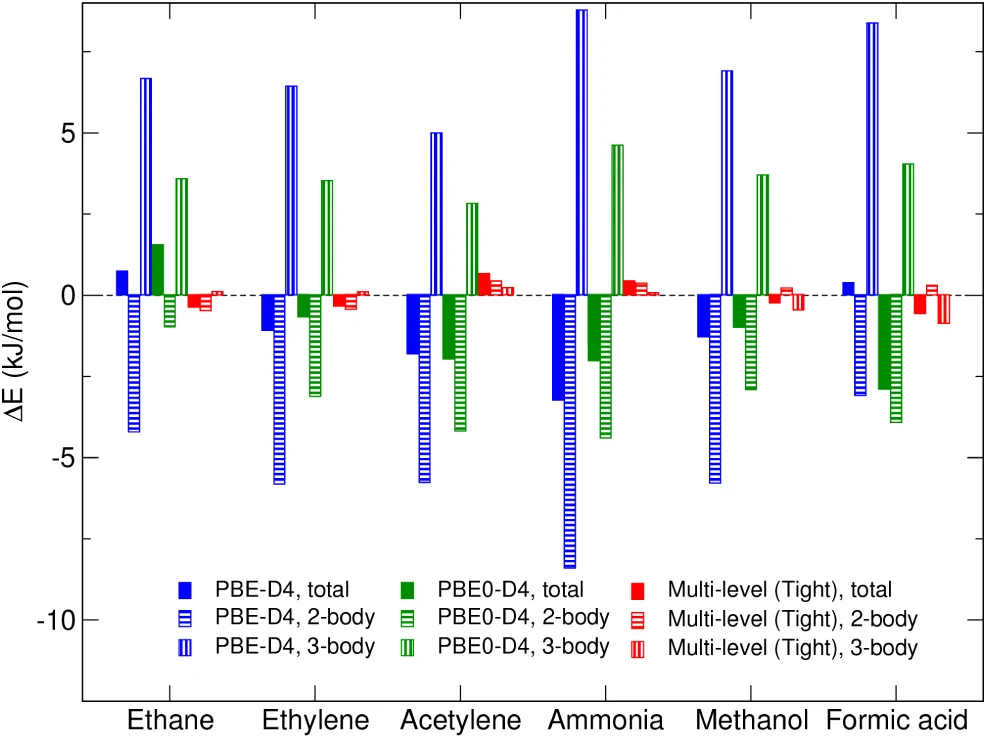
Errors of PBE-D4, PBE0-D4, and multi-level­(Tight) relative to the canonical CCSD­(T) reference.

By contrast, the multi-level LNO-based approach, even with the Tight variant, yields much smaller errors in the total energies and their two- and three-body components, with deviations of less than 0.9 kJ/mol from the reference (see Figure 12). This result supports the use of the LNO-CCSD­(T) multi-level scheme as a reference for calculating *n*-body energies and evaluating the performance of approximate methods.

## 4 Conclusions

We have investigated the effect of local approximations in the coupled-cluster treatment of the many-body expanded crystal lattice energies of small-molecule crystals. The presence of spurious long distance contributions appears for some crystals as the primary technical challenge. Namely, in all combinations of state-of-the-art local coupled-cluster methods with any of their pre-defined, off-the-shelf threshold sets, there are some cases where energy curves saturate but then start to drift or diverge in the correlation energy contribution when going to very long intermonomer distances.

Within the LNO-CCSD­(T) approach, which showed the most accurate and efficient performance, a thorough evaluation of the sources of errors points to the HF and LNO-CCSD iteration thresholds as well as natural auxiliary function truncations as the major source of numerical instability at very long intermolecular separations in the three-body contributions. Upon simultaneous tightening of the responsible thresholds in the LNO approximation, we find it possible to describe the pairwise and nonadditive three-body noncovalent interaction energies with numerical robustness at extremely long distances, with the cost still well below that of canonical calculations. The numerical precision of the introduced benchmark tier of LNO-CCSD­(T), referred to as vvTight+, is 0.2 kJ/mol (maximum error), which makes it indistinguishable from canonical CCSD­(T) in practical calculations of solids. Almost all updated LNO-CCSD­(T) precision settings will be a part of a forthcoming release of the Mrcc program suite.

For molecular crystals with large molecules, where the application of the highest precision settings is not realistic or does not warrant the cost-investment, we have proposed a similarly robust but more scalable multi-level protocol. In the two-body contribution to the multi-level lattice energy, the local-approximation-free extrapolation of LNO-CCSD­(T) gives excellent results. In the three-body contribution, the computationally optimal strategy is to match the LNO-CCSD­(T) description of compact trimers with the simplified, but extremely stable description of long-distance trimers with the random-phase approximation, making sure that the switchover radius precedes the onset of the energy drift. Overall, the multi-level method in the highest variant, vvTight, yields the total lattice energies with benchmark precision (maximum error of 0.2 kJ/mol). The lower variants of the multi-level approach are well-behaved at long range, systematically approach the reference, and have a tunable accuracy-to-cost ratio controlled by the LNO approximation.

The high efficiency and very low memory requirement of LNO-CCSD­(T) and its free and open availability for academic use, in combination with the massive parallelization potential of many-body expansions, make this approach practical for a broad computational community. We believe that our work providing a robust foundation for high precision and affordable computations, combined with the recent reports on the application of LNO-CCSD­(T) on the X23 dataset of solids and on even larger drug crystals,^15,31^ demonstrate the readiness of the local coupled-cluster methodology for generation of high-quality datasets of organic molecular solids. We note that this level of accuracy is established for the systems considered in the present work. For larger molecular crystals with significant conformational flexibility or large asymmetric units, the accuracy on the order of 1–2 kJ/mol is expected to remain realistic, although dedicated benchmarks are required to confirm this and are the subject of our ongoing work.

## Supplementary Material



## Data Availability

The data that support the findings of this study are available within the article, its supplementary material, and in the Zenodo repository (DOI: 10.5281/zenodo.20267257). The scripts used to generate the results are in the Zenodo repository. The Mrcc code is available at www.mrcc.hu and is free for academic use. The beyond-rpa program is available at https://github.com/modrzejewski/beyond-rpa.

## Notes

Tables of localized-orbital coupled-cluster accuracy settings, the effect of the PNO-LCCSD­(T) settings, two-body energy contributions, HF calculations, MP2 results, a comparison between results obtained using the 2024 and new LNO-CCSD­(T) settings, lattice energies of two forms of solid acetylene, and DFT results, together with figures illustrating the performance of localized-orbital coupled-cluster methods for two-body CCSD and (T) energy contributions, distance convergence of two- and three-body energy contributions, the performance of LNO-CCSD­(T) at short intermolecular distances and of the multi-level approach, CPU timings, and distance convergence of two forms of solid acetylene (PDF)
