# Supplementary material for: Sub-kJ/mol Precision at Practical Cost for Molecular Solids through Local Coupled-Cluster Approaches
Source: J Phys Chem A. 2026 Oct 14;130(43):8707–21. doi: 10.1021/acs.jpca.6c05975 (PMC13726384; doi:10.1021/acs.jpca.6c05975)
Supplement: Supplementary file 1 [file jp6c05975_si_001.pdf]

Supporting Information for "Sub-kJ/mol Precision at Practical Cost for Molecular Solids through Local Coupled Cluster Approaches"

Khanh Ngoc Pham[1], Marcin Modrzejewski[2], Péter R. Nagy[3], and Jiří Klimeš[1]

[1]Department of Chemical Physics and Optics, Faculty of Mathematics and Physics, Charles University, Ke Karlovu 3, CZ-12116 Prague 2, Czech Republic

[2]Faculty of Chemistry, University of Warsaw, Pasteura 1, 02-093 Warsaw, Poland

[3]Department of Physical Chemistry and Materials Science, Faculty of Chemical Technology and Biotechnology, Budapest University of Technology and Economics, Műegyetem rkp. 3., H-1111 Budapest, Hungary

[3]HUN-REN–BME Quantum Chemistry Research Group, Műegyetem rkp. 3., H-1111 Budapest, Hungary

[3]MTA–BME Lendület Quantum Chemistry Research Group, Műegyetem rkp. 3., H-1111 Budapest, Hungary

Contents

1 **Technical details** **S3**

2 **Effect of the PNO-LCCSD(T) settings** **S5**

3 **Hartree-Fock calculations** **S6**

4 **Two-body energy contributions** **S7**

5 Convergence with distance **S12**
5.1 PNO-LCCSD(T) S12
5.2 DLPNO-CCSD(T) S13
5.3 LNO-CCSD(T) S16
5.4 Multi-level approach S21
6 MP2 results **S22**
7 Accuracy of LNO-CCSD(T) at $R < 17$ Å **S23**
8 Accuracy of the multi-level approach **S24**
9 CPU timings **S25**
10 Polymorphs of acetylene **S27**
11 DFT results **S30**
12 Geometry input file for PBC calculations **S31**

1 Technical details

Table S1: Accuracy settings for PNO coupled-cluster calculations. The given thresholds are taken directly from the manual for local correlation methods with pair natural orbitals in Molpro.

Threshold	Default	Tight	vTight
	DOMOPT		
**PAO domains**			
IEXT	2	3	3
REXT/a_0	5	7	7
**PNO_LMP2 domains**			
THRPNO_LMP2	10^{-8}	10^{-8}	10^{-9}
THREN_LMP2	0.997	0.997	0.997
**PNO_LCCSD domains**			
THRPNO_CC	10^{-7}	10^{-8}	2.5×10^{-9}
THREN_CC	0.990	0.990	0.997
**triples domains**			
THRTNO_T0	10^{-9}	10^{-10}	10^{-10}
THRTNO_T	10^{-7}	10^{-7}	5×10^{-8}
	PAIROPT		
**pair lists**			
THRCLOSE	10^{-4}	10^{-5}	
THRWEAK	10^{-5}	10^{-6}	
THRDIST	10^{-6}	10^{-6}	
THRDISTC	10^{-6}	10^{-6}	
**triples list**			
THRCLOSE_T_0	10^{-4}	10^{-5}	
THRTRIP_T	10^{-7}	10^{-8}	

Table S2: Accuracy settings for DLPNO coupled cluster. The given thresholds are taken directly from the ORCA Manual, Release 6.0 (version 6.0.1).

Threshold	LoosePNO	NormalPNO	TightPNO
$T_{CutPairs}$	10^{-3}	10^{-4}	10^{-5}
T_{CutDO}	2×10^{-2}	1×10^{-2}	5×10^{-3}
T_{CutPNO}	10^{-6}	3.33×10^{-7}	10^{-7}
T_{CutMKN}	10^{-3}	10^{-3}	10^{-3}
MP2 pair treatmeant	semicanonical	semicanonical	full iterative

Table S3: Accuracy settings for LNO coupled cluster in MRCC (version 2025.1.0).

Threshold	Normal	Tight	vTight	vvTight
bpedo	0.9999	0.99995	0.99999	0.999995
wpairtol	1×10^{-5}	3×10^{-6}	1×10^{-6}	3×10^{-7}
lnoepso	1×10^{-5}	3×10^{-6}	1×10^{-6}	3×10^{-7}
lnoepsv	1×10^{-6}	3×10^{-7}	1×10^{-7}	3×10^{-8}
naf_cor	1×10^{-2}	1×10^{-2}	1×10^{-3}	1×10^{-3}

2 Effect of the PNO-LCCSD(T) settings

Table S4: Precision of pair approximations in PNO-LCCSD(T) calculations for 2-body and 3-body energies of ethylene and ammonia. All values are reported in kJ/mol using the AVTZ basis set.

Systems	Contributions	DOMOPT options	PAIROPT options	PNO-LCCSD	PNO-L(T)	PNO-LCCSD(T)
Ethylene	2-body	Tight	Default	−26.86	−3.89	−30.75
			Tight	−26.60	−3.84	−30.44
		vTight	Default	−26.99	−4.14	−31.13
			Tight	−26.71	−4.09	−30.80
Ammonia	2-body	Tight	Default	−20.34	−3.37	−23.71
			Tight	−20.17	−3.33	−23.50
		vTight	Default	−20.46	−3.58	−24.04
			Tight	−20.28	−3.54	−23.82
	3-body	Tight	Default	1.44	0.43	1.87
			Tight	1.39	0.38	1.77
		vTight	Default	1.11	0.09	1.20
			Tight	1.11	0.09	1.20

3 Hartree-Fock calculations

Table S5: Two-body and three-body Hartree–Fock (HF) energies (kJ/mol), computed using the AVQZ and AVTZ basis sets, respectively.

Contribution	Ethane	Ethylene	Acetylene	Ammonia	Methanol	Formic acid
2-body	12.95	9.38	−2.24	−10.68	−10.52	−40.98
3-body	−0.61	−0.38	−0.16	−0.67	−7.61	−4.31
Total (2-body + 3-body)	12.34	9.00	−2.40	−11.35	−18.13	−45.29

Table S6: The *k*-point set and simulation box ($a \times a \times a$ Å^3) used to obtain the energies of solid and molecule, respectively, along with the HF binding energies ($E_{\text{bind}}^{\text{HF}}$, kJ/mol).

Systems	*k*-points	a	$E_{\text{bind}}^{\text{HF}}$
Ethane	$6 \times 6 \times 6$	25	12.35
Ethylene	$6 \times 6 \times 6$	25	8.89
Acetylene	$3 \times 3 \times 3$	25	−2.55
Ammonia	$4 \times 4 \times 4$	25	−11.09
Methanol	$4 \times 4 \times 4$	25	−18.12
Formic acid	$3 \times 7 \times 4$	25	−48.18

4 Two-body energy contributions

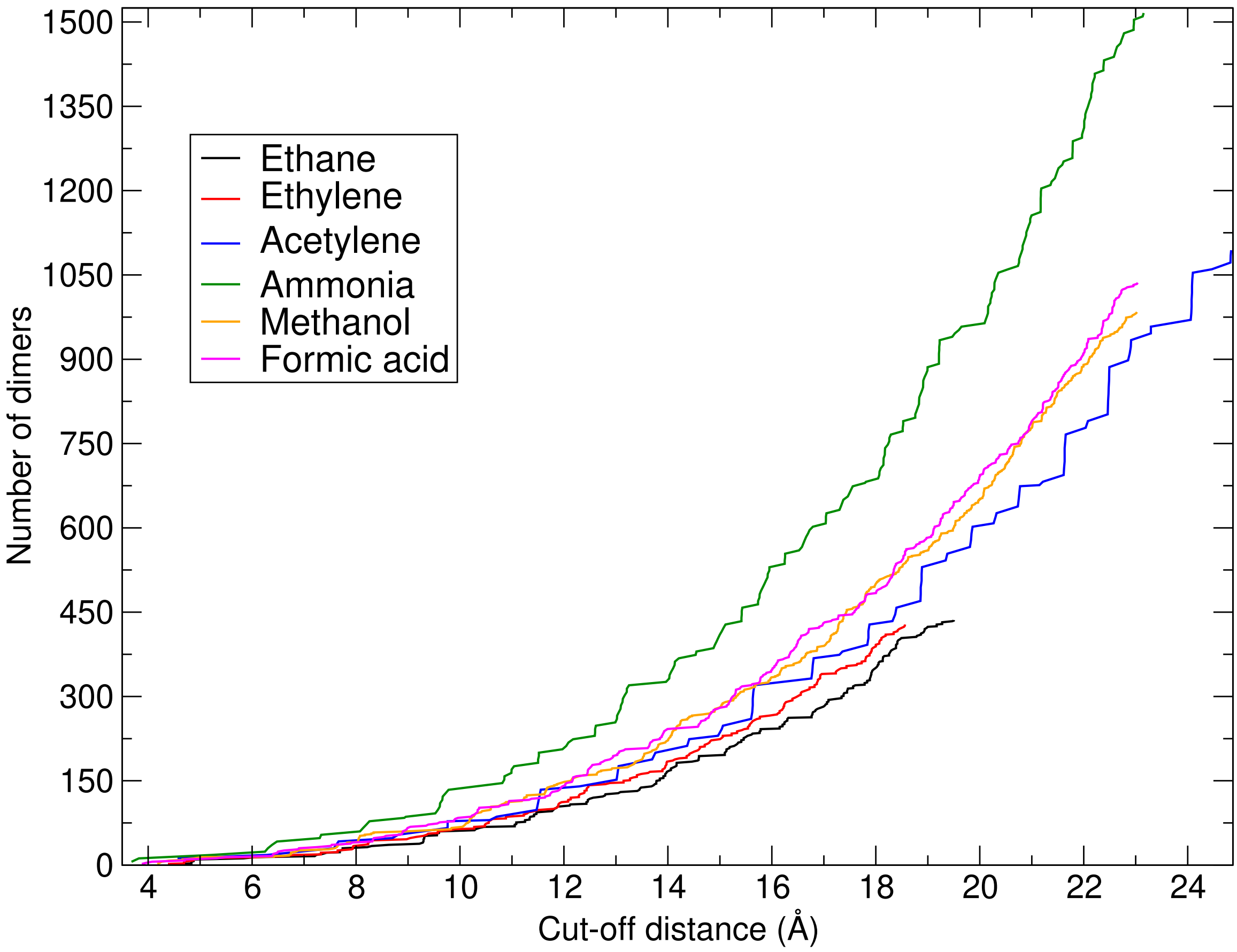

Figure S1: Number of dimers as a function of cut-off distance for all the considered systems.

Table S7: The two-body CCSD/CBS correlation energy contributions (kJ/mol) obtained from reference CCSD(T) and localized-orbital coupled cluster approaches for all the considered systems. We note that in the presence of numerical noise, the last stable value is retained and the final energy is estimated from distance convergence behavior.

Threshold	Ethane	Ethylene	Acetylene	Ammonia	Methanol	Formic acid
			Canonical CCSD			
–	−31.20	−27.52	−19.42	−21.60	−32.20	−32.23
			PNO-LCCSD			
Tight	−31.10	−27.63	−19.79	−21.50	−31.92	−32.10
vTight	−31.13	−27.70	−19.78	−21.54	−32.00	−32.34
			DLPNO-CCSD			
Normal	−32.57	−30.82	−23.82	−21.63	−32.26	−33.22
Tight	−31.79	−28.29	−20.67	−21.58	−32.28	−32.82
			LNO-CCSD			
Normal	−27.65	−25.63	−18.59	−19.02	−27.32	−25.05
Tight	−29.67	−26.74	−19.17	−20.79	−30.10	−29.36
vTight	−30.63	−27.36	−19.56	−21.16	−31.34	−31.27
vvTight	−31.16	−27.68	−19.77	−21.58	−32.08	−32.09

Table S8: The two-body (T)/CBS correlation energy contributions (kJ/mol) obtained from reference CCSD(T) and localized-orbital coupled cluster approaches for all the considered systems. We note that in the presence of numerical noise, the last stable value is retained and the final energy is estimated from distance convergence behavior.

Threshold	Ethane	Ethylene	Acetylene	Ammonia	Methanol	Formic acid
			Canonical (T)			
–	−5.61	−5.39	−4.28	−4.33	−6.44	−7.73
			PNO-L(T)			
Tight	−4.31	−3.89	−2.85	−3.46	−4.93	−5.19
vTight	−4.59	−4.16	−3.10	−3.69	−5.26	−5.89
			DLPNO-(T)			
Normal	−1.96	−1.76	−1.33	−1.85	−2.15	−0.95
Tight	−4.41	−4.08	−3.15	−3.34	−4.64	−4.68
			LNO-(T)			
Normal	−7.25	−7.10	−6.54	−6.24	−9.04	−12.28
Tight	−6.76	−6.32	−4.76	−4.66	−7.52	−9.31
vTight	−5.94	−5.76	−4.67	−4.53	−6.78	−8.34
vvTight	−5.72	−5.50	−4.37	−4.35	−6.59	−7.89

Table S9: The total two-body CCSD(T)/CBS energies (kJ/mol) obtained from canonical CCSD(T) and local-orbital coupled-cluster approaches for all considered systems. We note that in the presence of numerical noise, the last stable value is retained and the final energy is estimated from distance convergence behavior. The HF energies were taken from Table S5.

Threshold	Ethane	Ethylene	Acetylene	Ammonia	Methanol	Formic acid
			Canonical CCSD(T)			
–	−23.86	−23.53	−25.95	−36.61	−49.16	−80.94
			PNO-LCCSD(T)			
Tight	−22.46	−22.14	−24.88	−35.64	−47.37	−78.27
vTight	−22.77	−22.48	−25.12	−35.91	−47.78	−79.21
			DLPNO-CCSD(T)			
Normal	−21.59	−23.20	−27.39	−34.16	−44.93	−75.15
Tight	−23.25	−22.99	−26.07	−35.60	−47.44	−78.47
			LNO-CCSD(T)			
Normal	−21.95	−23.35	−27.37	−35.95	−46.88	−78.31
Tight	−23.48	−23.68	−26.17	−36.13	−48.14	−79.65
vTight	−23.62	−23.74	−26.46	−36.37	−48.64	−80.59
vvTight	−23.93	−23.80	−26.37	−36.62	−49.19	−80.96

Table S10: Energy difference between the 2024 and new settings for two-body LNO-CCSD(T) correlation energy of the considered systems (kJ/mol).

Threshold	Ethane	Ethylene	Acetylene	Ammonia	Methanol	Formic acid
Normal	0.13	0.02	0.04	0.09	0.18	0.28
Tight	0.02	0.08	0.03	0.03	0.05	0.12
vTight	0.03	0.04	0.02	0.00	0.09	0.06
vvTight	0.01	0.03	0.10	0.02	0.01	0.01

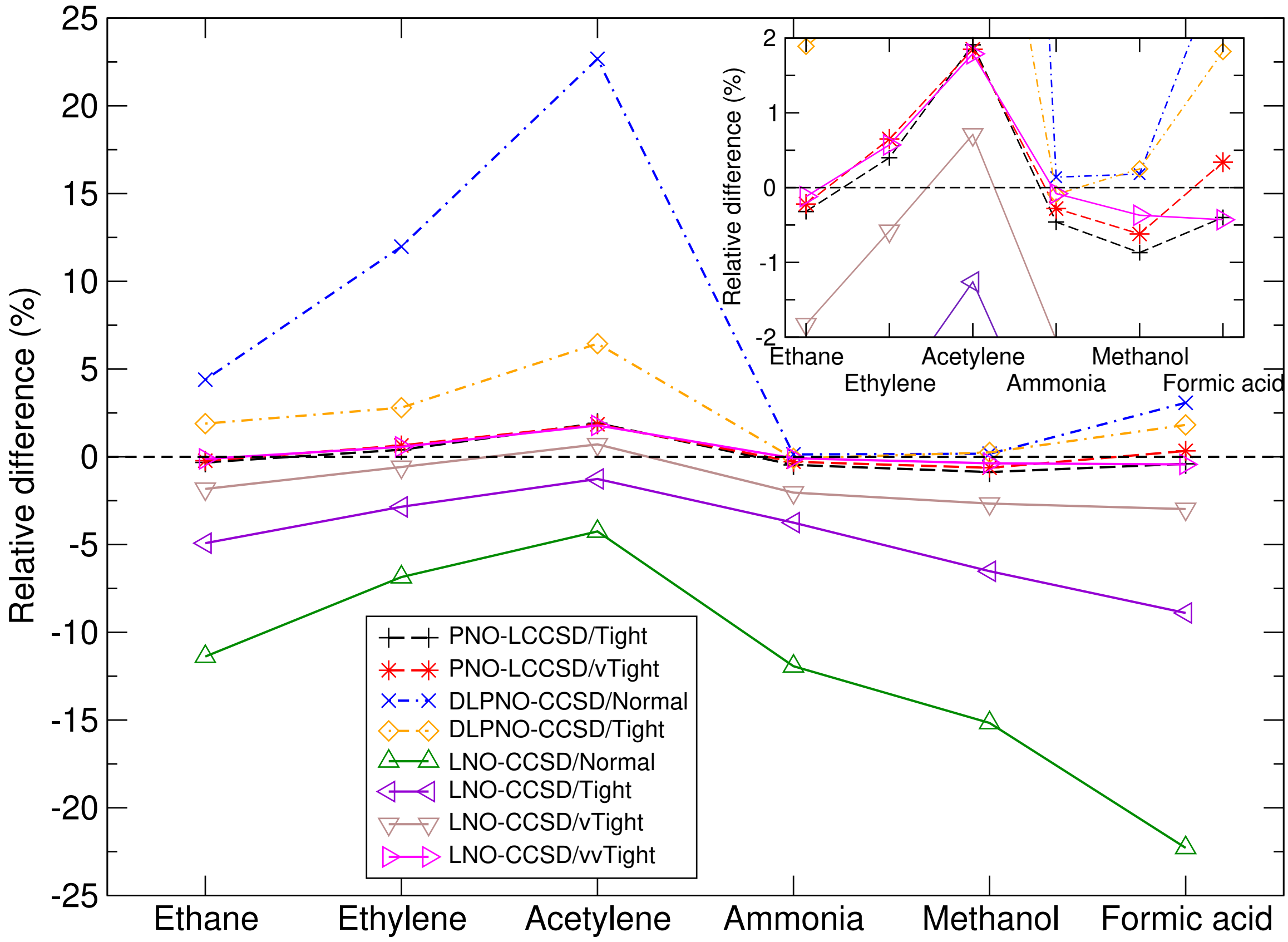

Figure S2: The performance of localized orbital coupled-cluster methods for the two-body CCSD correlation energy.

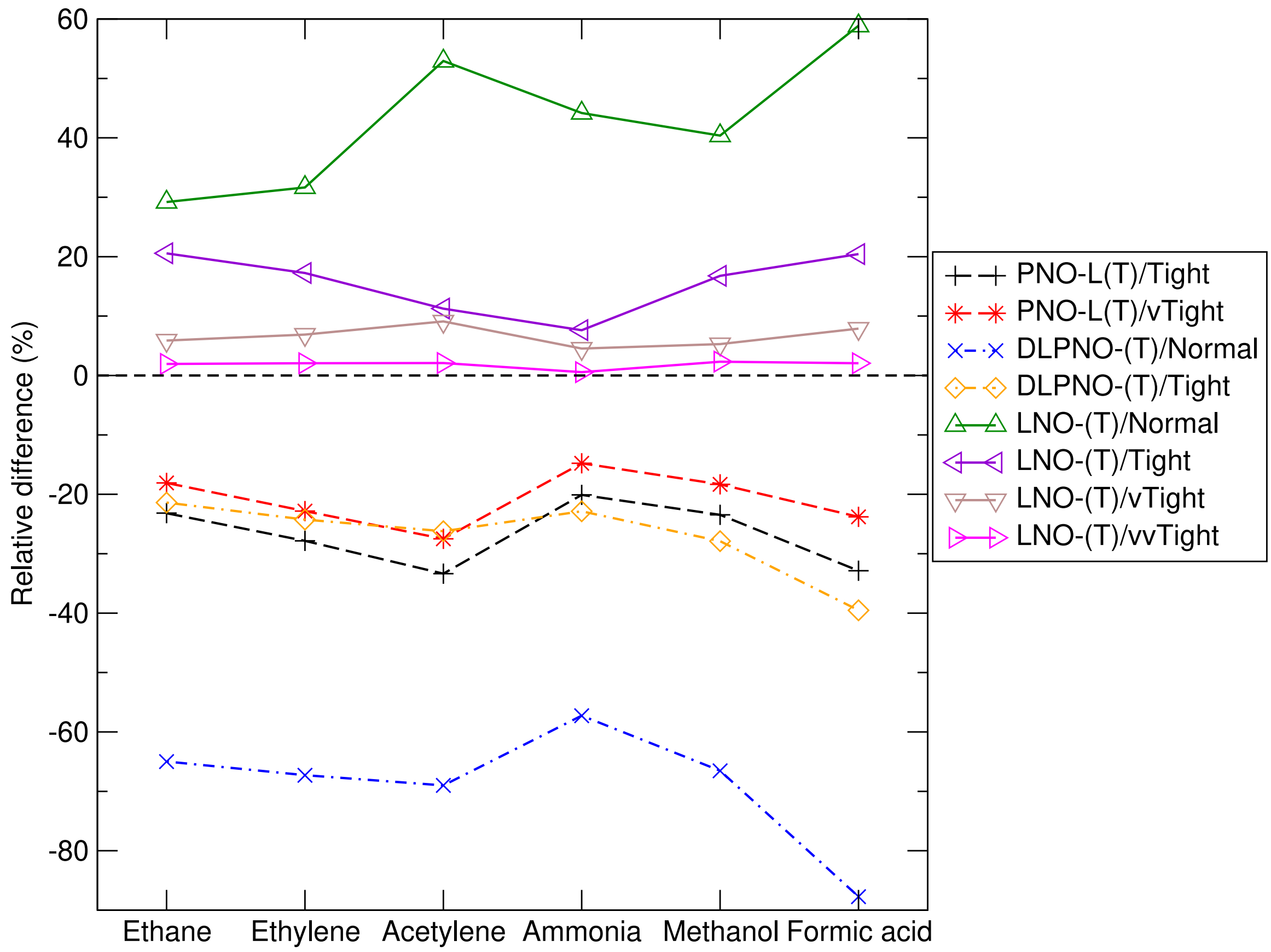

Figure S3: The performance of localized orbital coupled-cluster methods for the two-body (T) correlation energy.

5 Convergence with distance

5.1 PNO-LCCSD(T)

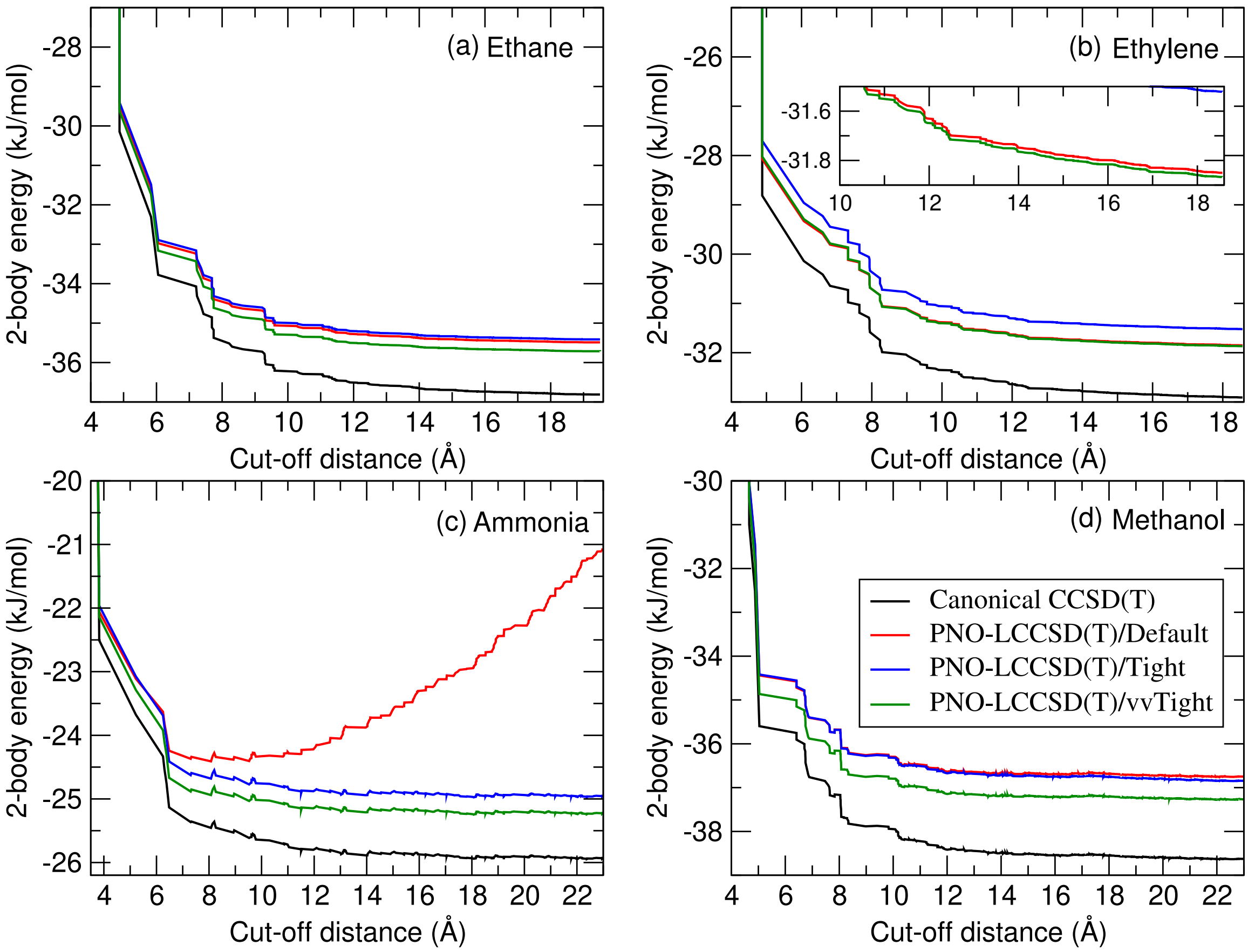

Figure S4: The distance convergence of the two-body PNO-LCCSD(T) correlation energy for (a) ethane, (b) ethylene, (c) ammonia, and (d) ammonia. The data were calculated with AVTZ→AVQZ extrapolation. We note that the distance convergence of the two-body PNO-LCCSD(T) correlation energy of formic acid is similar to that of ammonia.

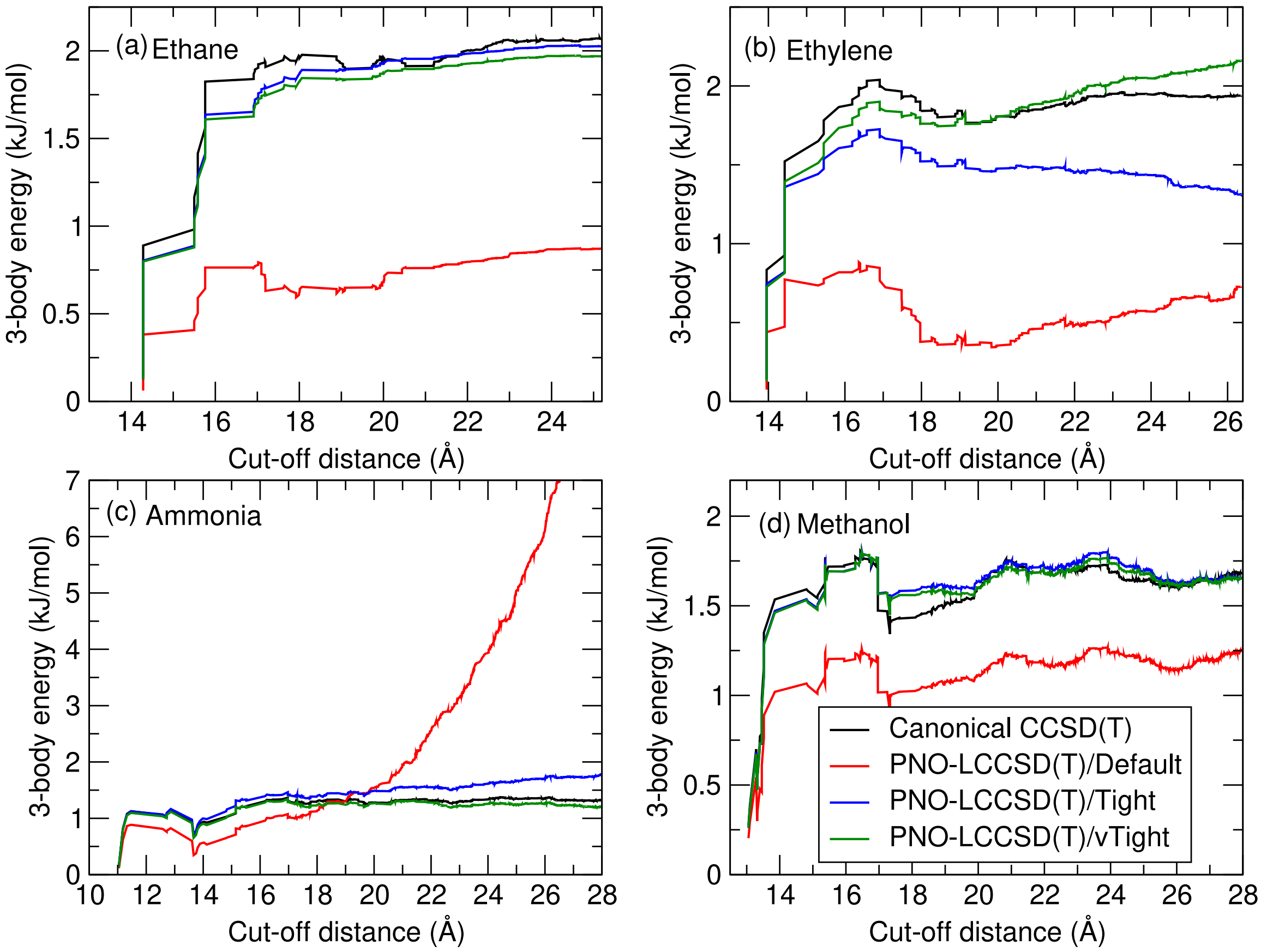

Figure S5: The distance convergence of the three-body PNO-LCCSD(T) correlation energy for (a) ethane, (b) ethylene, (c) ammonia, and (d) methanol. The data were calculated with AVTZ basis set. We note that the distance convergence of the three-body PNO-LCCSD(T) correlation energy of formic acid is similar to that of methanol.

5.2 DLPNO-CCSD(T)

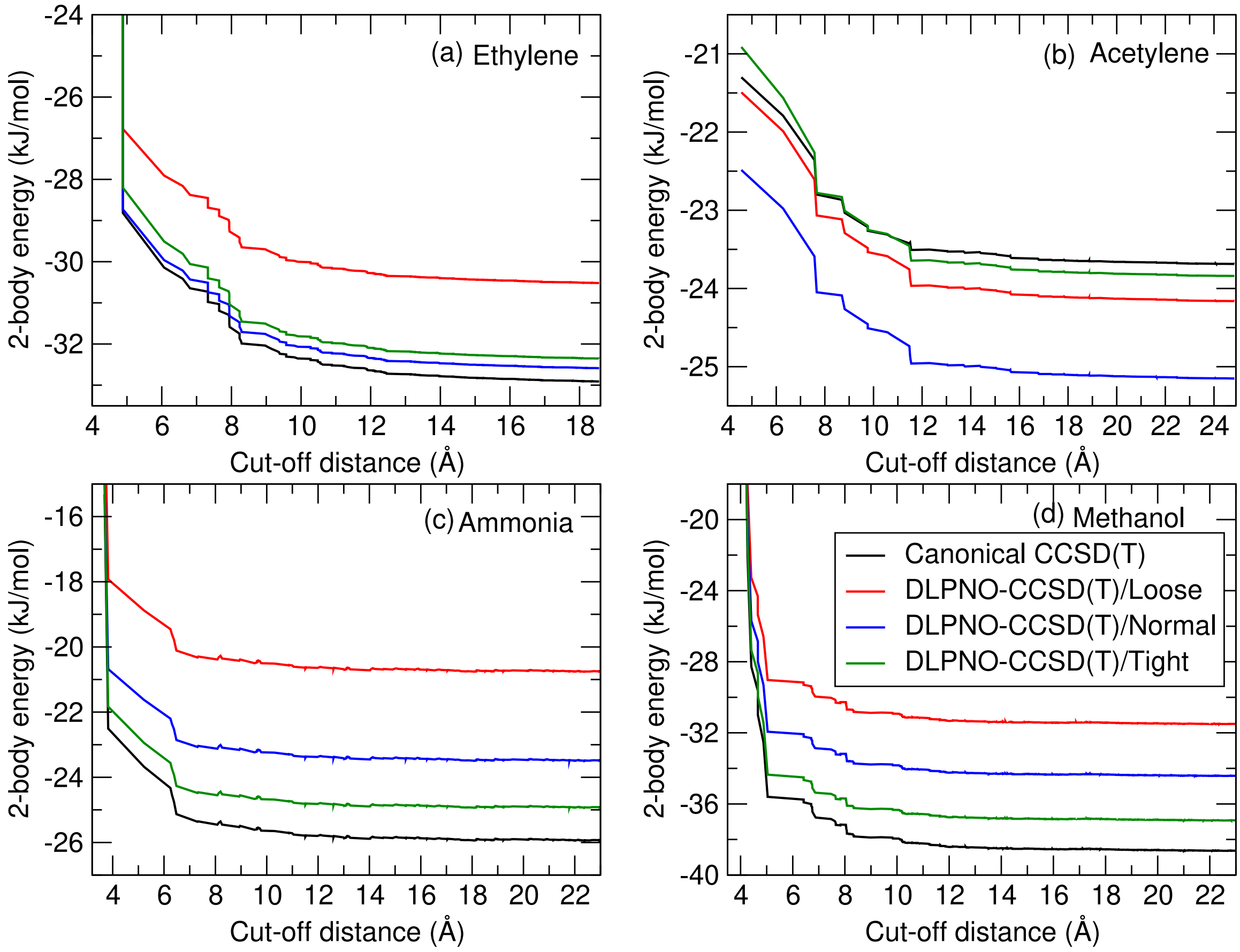

Figure S6: The distance convergence of the two-body DLPNO-CCSD(T) correlation energy for (a) ethylene, (b) acetylene, (c) ammonia, and (d) methanol. The data were calculated with AVTZ→AVQZ extrapolation. We note that the distance convergence of the two-body DLPNO-CCSD(T) correlation energy of ethane is similar to that of ethylene and acetylene.

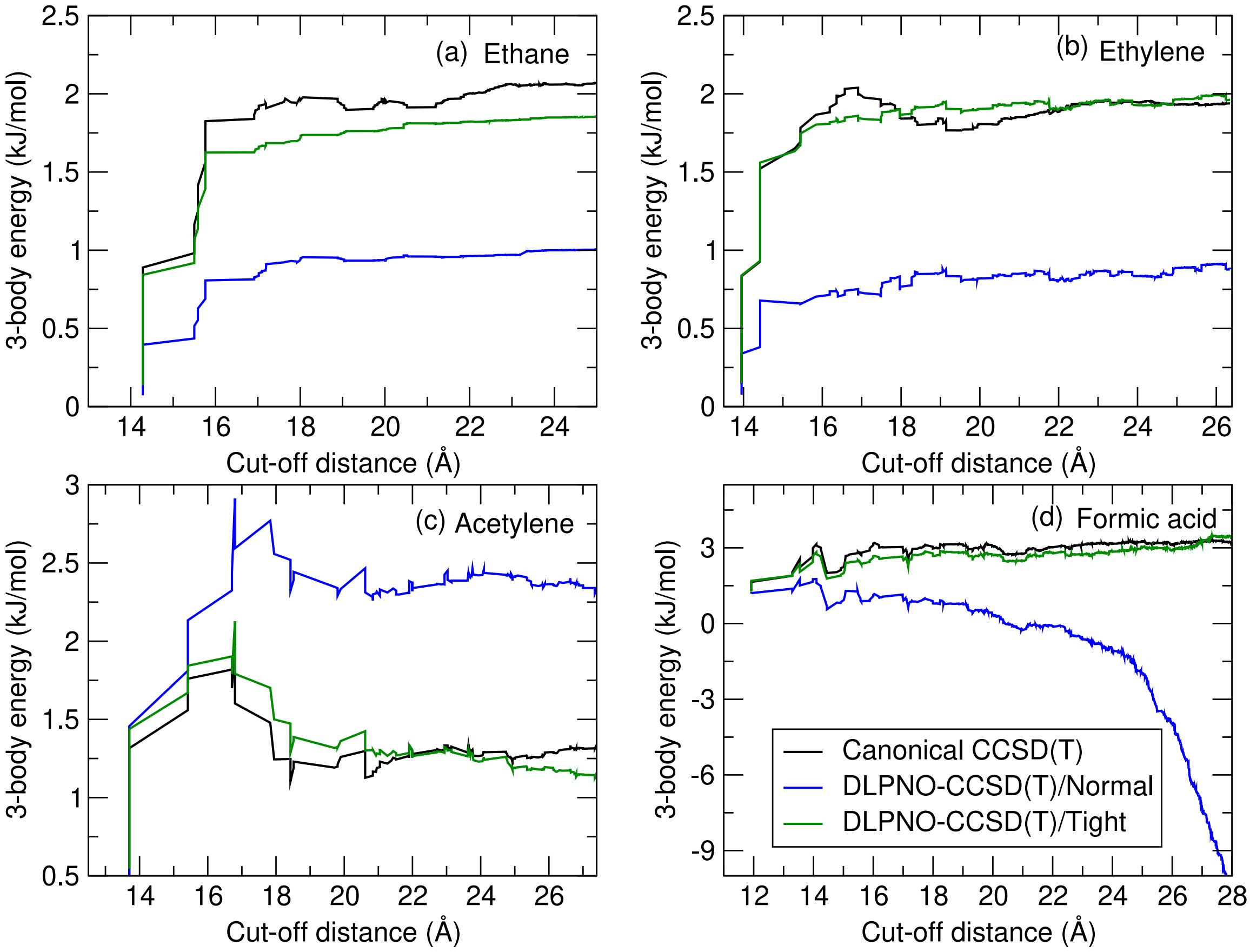

Figure S7: The distance convergence of the three-body DLPNO-CCSD(T) correlation energy for (a) ethane, (b) ethylene, (c) acetylene, and (d) formic acid. The data were calculated with AVTZ basis set. We note that the distance convergence of the three-body DLPNO-CCSD(T) correlation energy of ammonia is similar to that of formic acid.

5.3 LNO-CCSD(T)

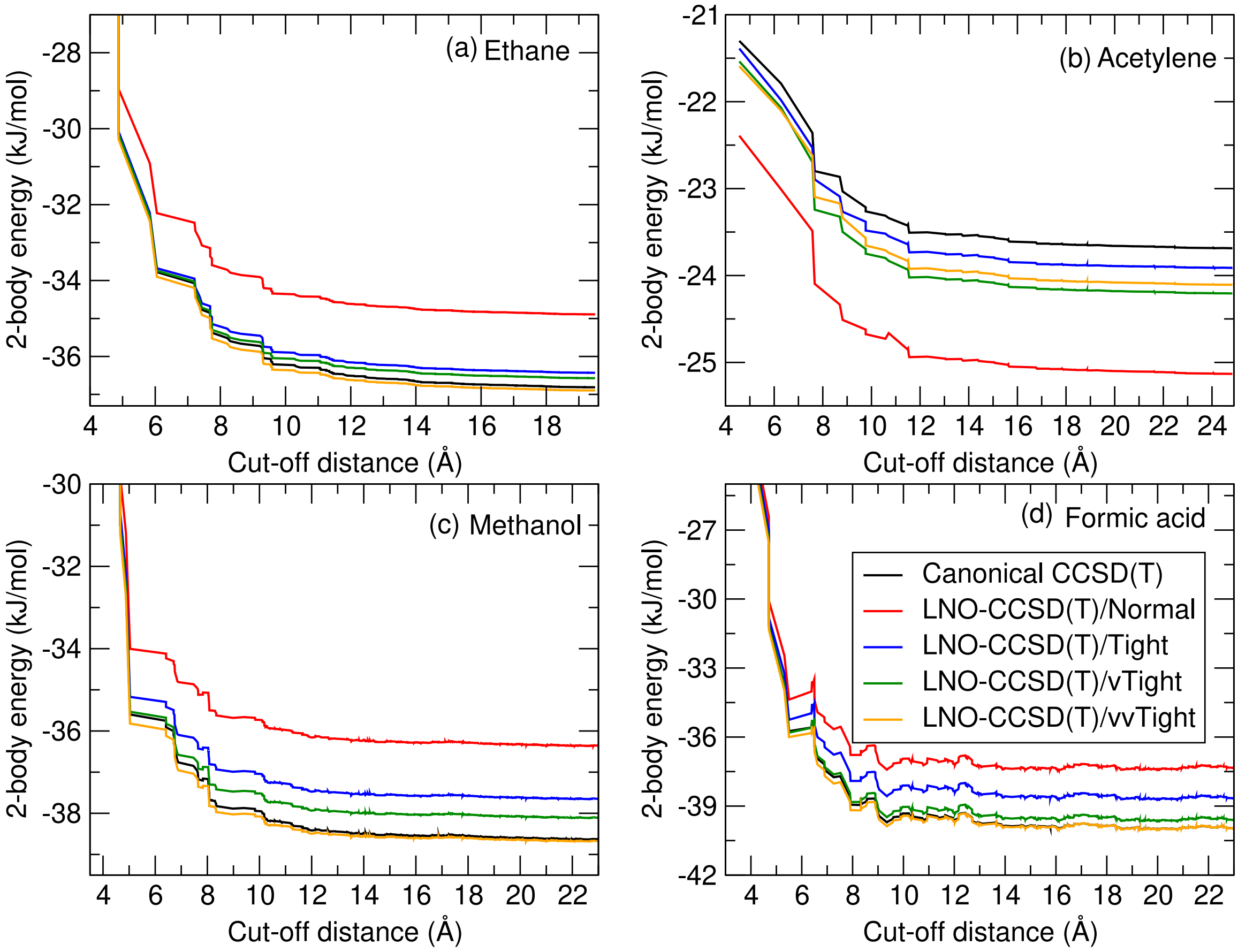

Figure S8: The distance convergence of the two-body LNO-CCSD(T) correlation energy for (a) ethane, (b) acetylene, (c) methanol, and (d) formic acid. The data were calculated with AVTZ→AVQZ extrapolation.

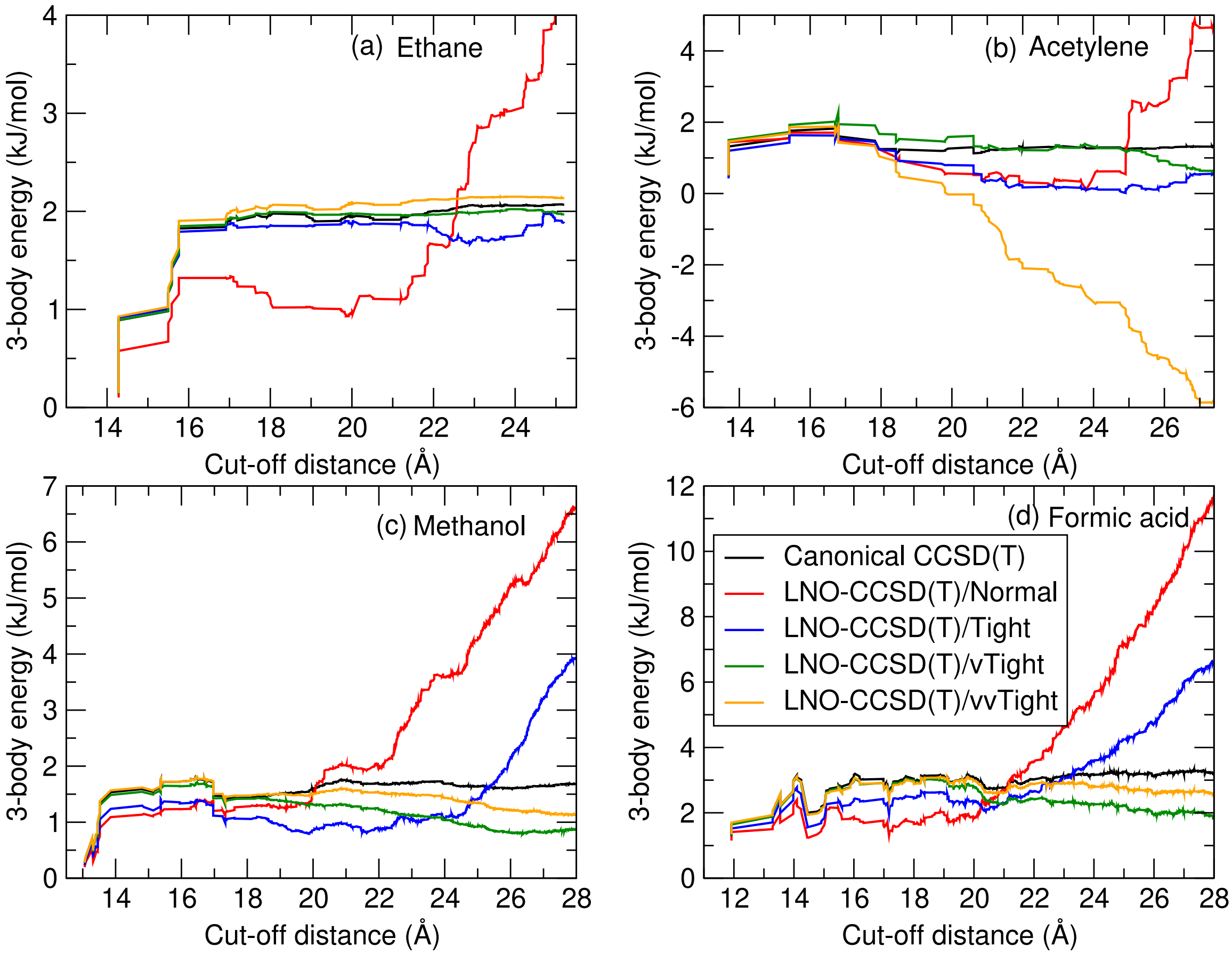

Figure S9: The distance convergence of the three-body LNO-CCSD(T) correlation energy for (a) ethane, (b) acetylene, (c) methanol, and (d) formic acid. The data were calculated with AVTZ basis set.

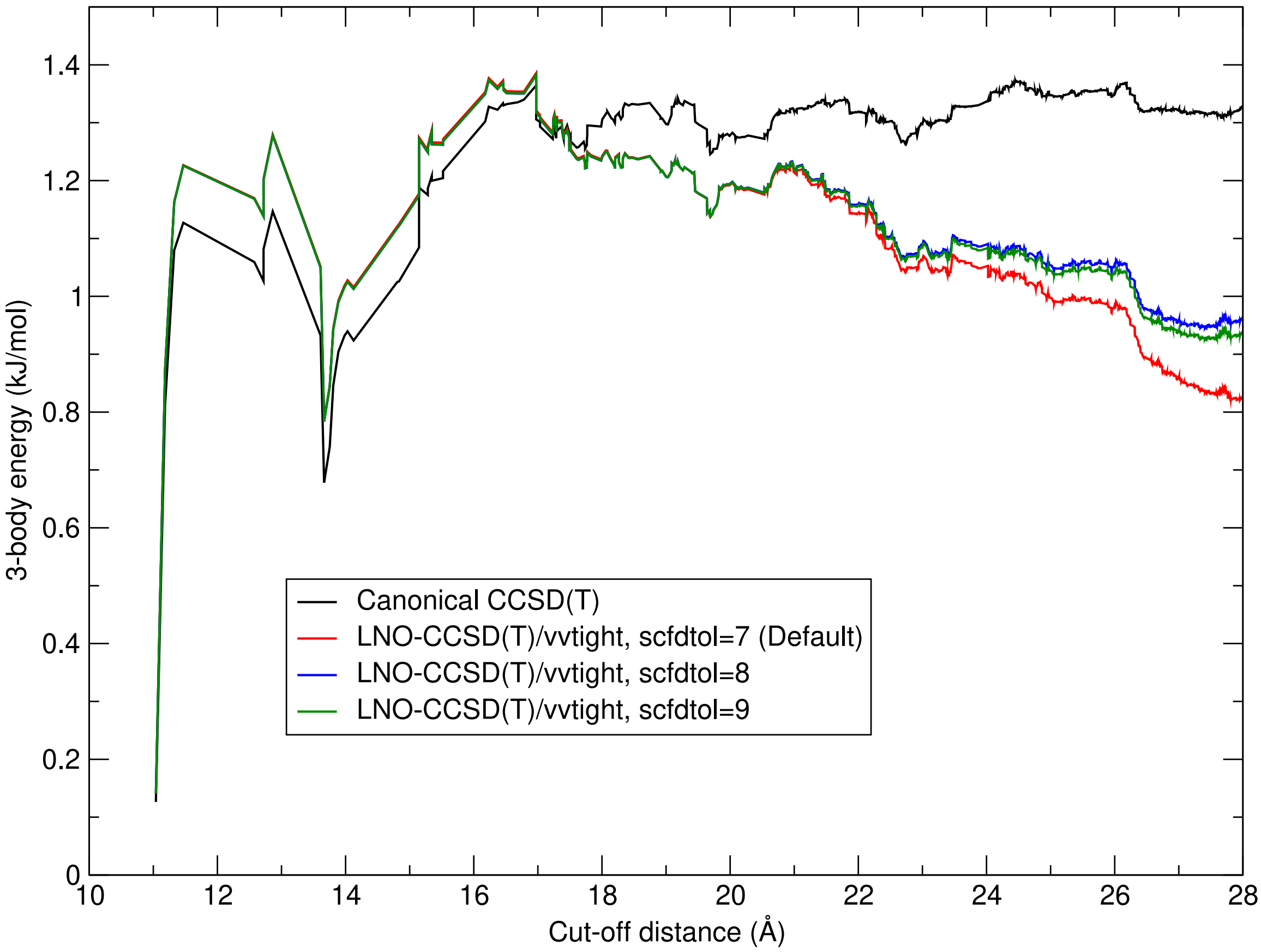

Figure S10: The distance convergence of the three-body LNO-CCSD(T) correlation energy of ammonia using different SCF settings. The data were calculated with AVTZ basis set.

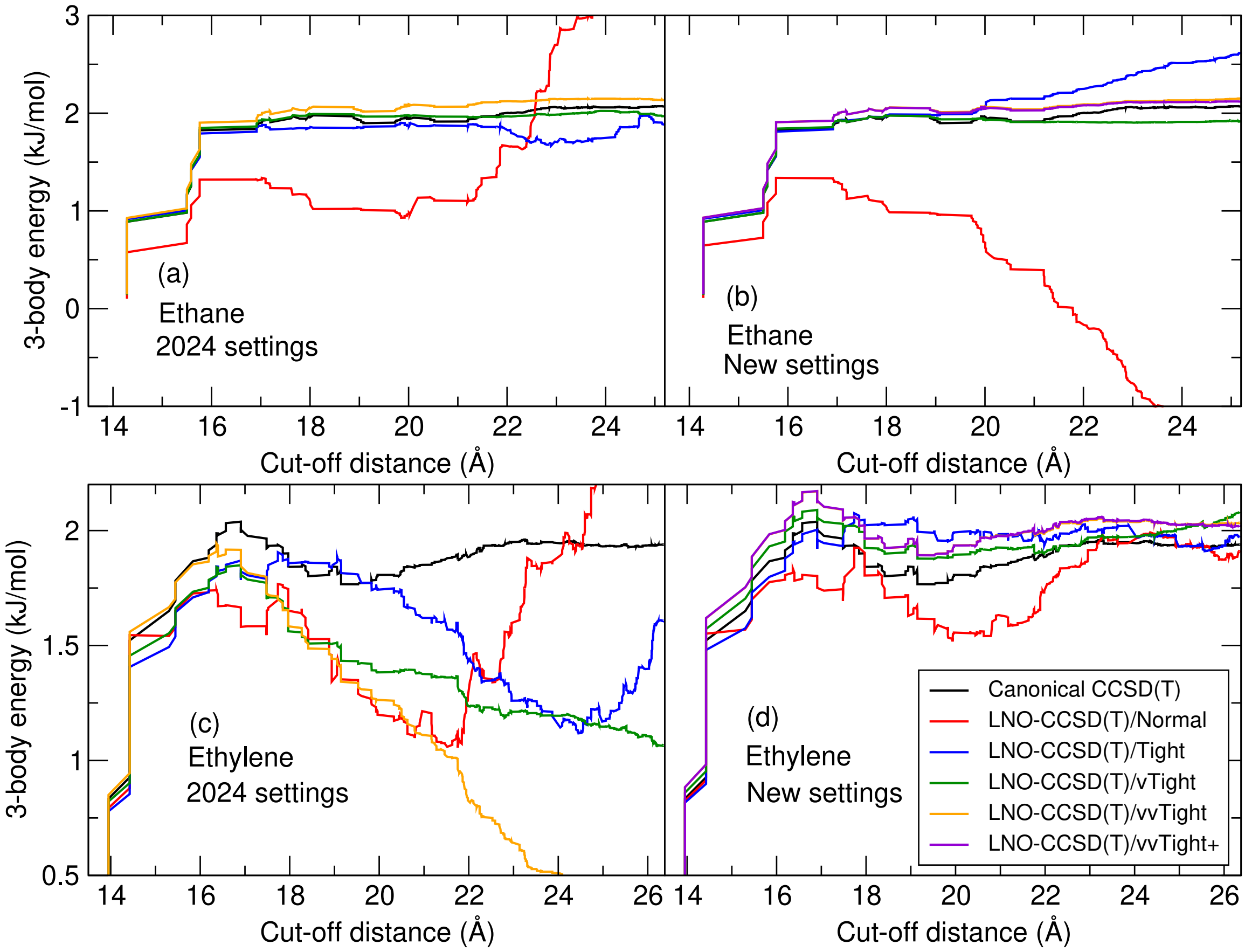

Figure S11: Distance convergence of the LNO-CCSD(T) correlation contribution to the three-body energy: (a) ethane with the 2024 settings, (b) ethane with the new settings, (c) ethylene with the 2024 settings, and (d) ethylene with the new settings. The LNO-CCSD(T)/vvTight and LNO-CCSD(T)/vvTight+ curves overlap in the case of new settings.

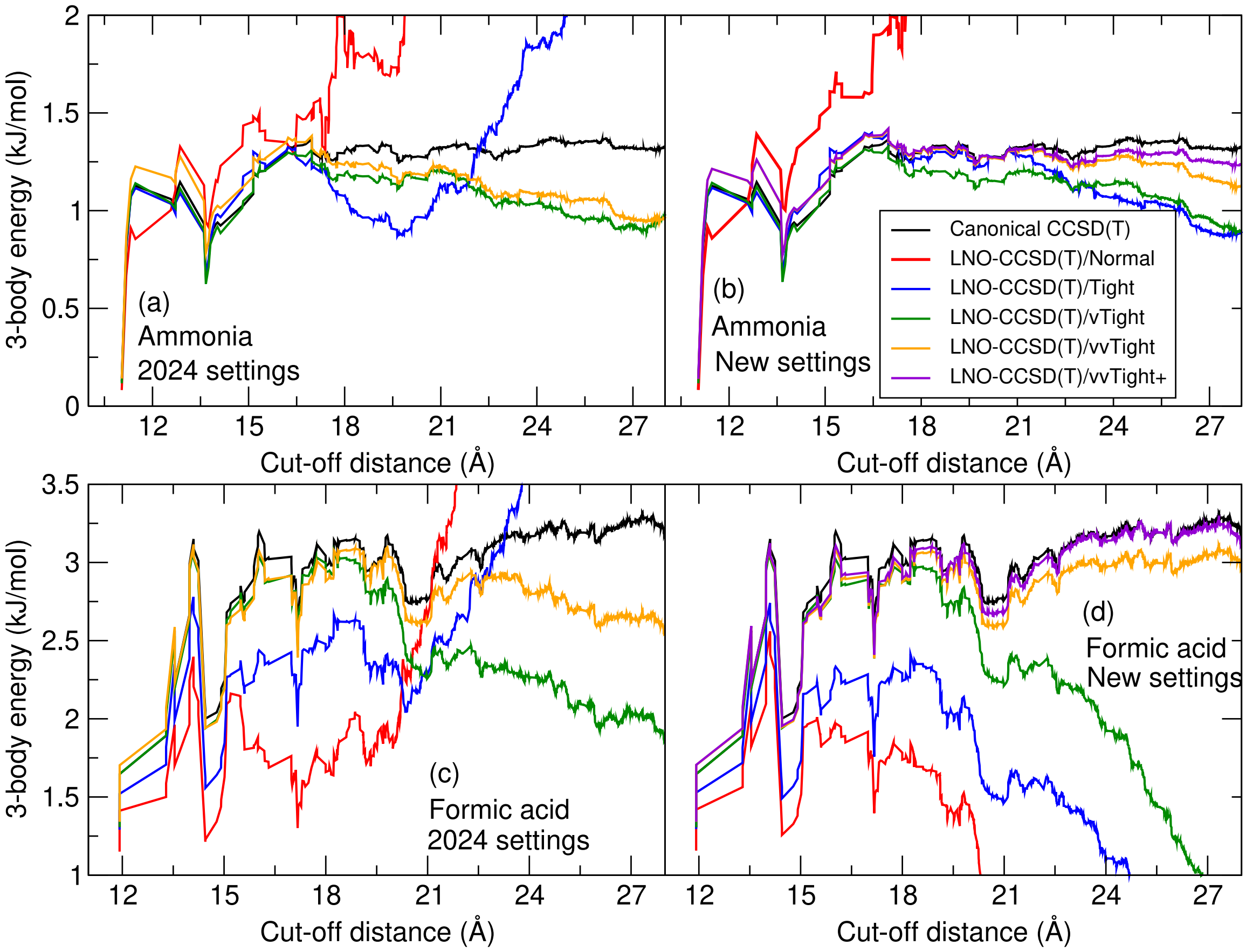

Figure S12: Distance convergence of the LNO-CCSD(T) correlation contribution to the three-body energy: (a) ammonia with the 2024 settings, (b) ammonia with the new settings, (c) formic acid with the 2024 settings, and (d) formic acid with the new settings. We note that the LNO-CCSD(T)/vvTight and canonical CCSD(T) curves almost overlap in the case of new settings of formic acid.

5.4 Multi-level approach

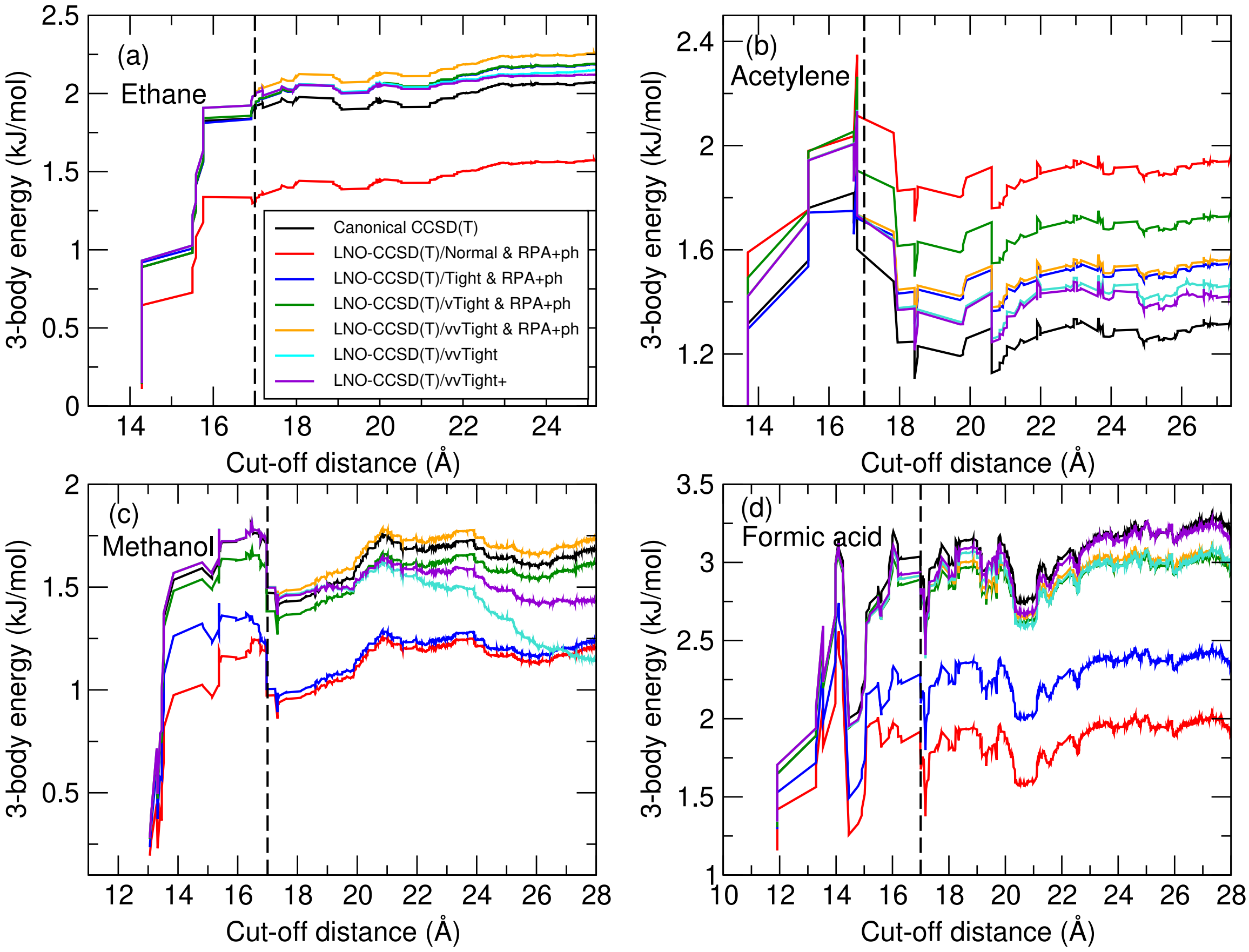

Figure S13: Distance convergence of the correlation contribution to the three-body energy in the multi-level approach for (a) ethane, (b) acetylene, (c) methanol, and (d) formic acid. The vertical bar marks the transition from LNO-CCSD(T) to RPA+ph. LNO-CCSD(T) employs the updated thresholds from Table 2 in the main text.

6 MP2 results

Table S11: Two-body and three-body MP2 energies (kJ/mol). The two-body correlation energies were obtained by CBS extrapolation using the AVTZ and AVQZ basis sets. The three-body correlation energies were computed using the AVTZ basis set. The HF energies were taken from Table S5.

Method	Contribution	Ethane	Ethylene	Acetylene	Ammonia	Methanol	Formic acid
Canonical MP2	2-body	−22.85	−25.66	−29.66	−34.88	−47.59	−78.11
	3-body	0.04	0.42	0.44	−0.24	−7.12	−2.80
Local MP2	2-body(vTight)	−22.64	−25.45	−29.54	−35.05	−47.53	−78.45
	2-body(vvTight)	−22.74	−25.54	−29.58	−35.08	−47.89	−78.59
	3-body(vvTight)	0.05	0.42	0.54	−0.36	−7.53	−2.84
	3-body(vvTight+)	0.02	0.40	0.49	−0.24	−7.23	−2.70

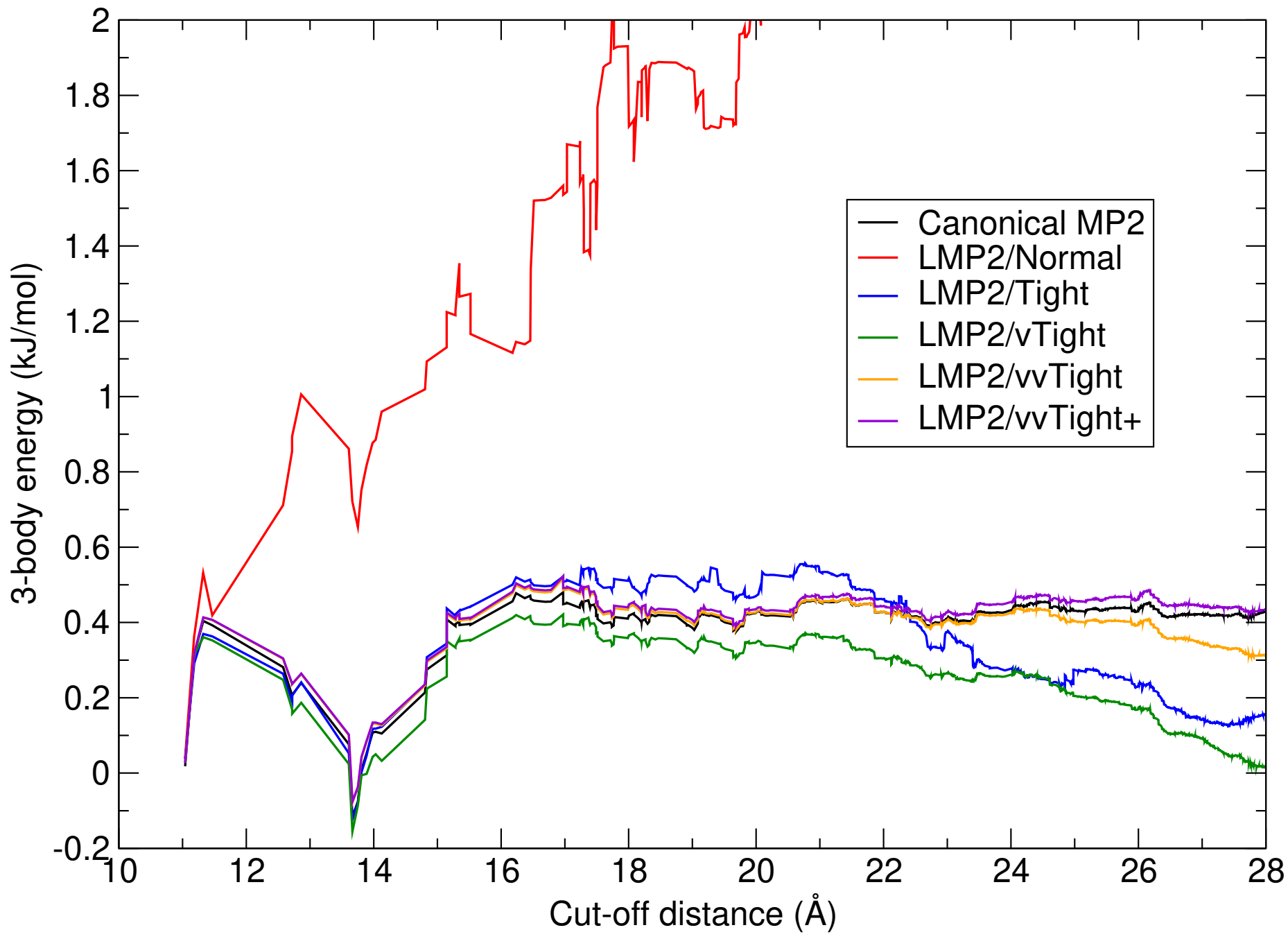

Figure S14: Distance convergence of the MP2 correlation contribution to the three-body energy for ammonia.

7 Accuracy of LNO-CCSD(T) at $R < 17$ Å

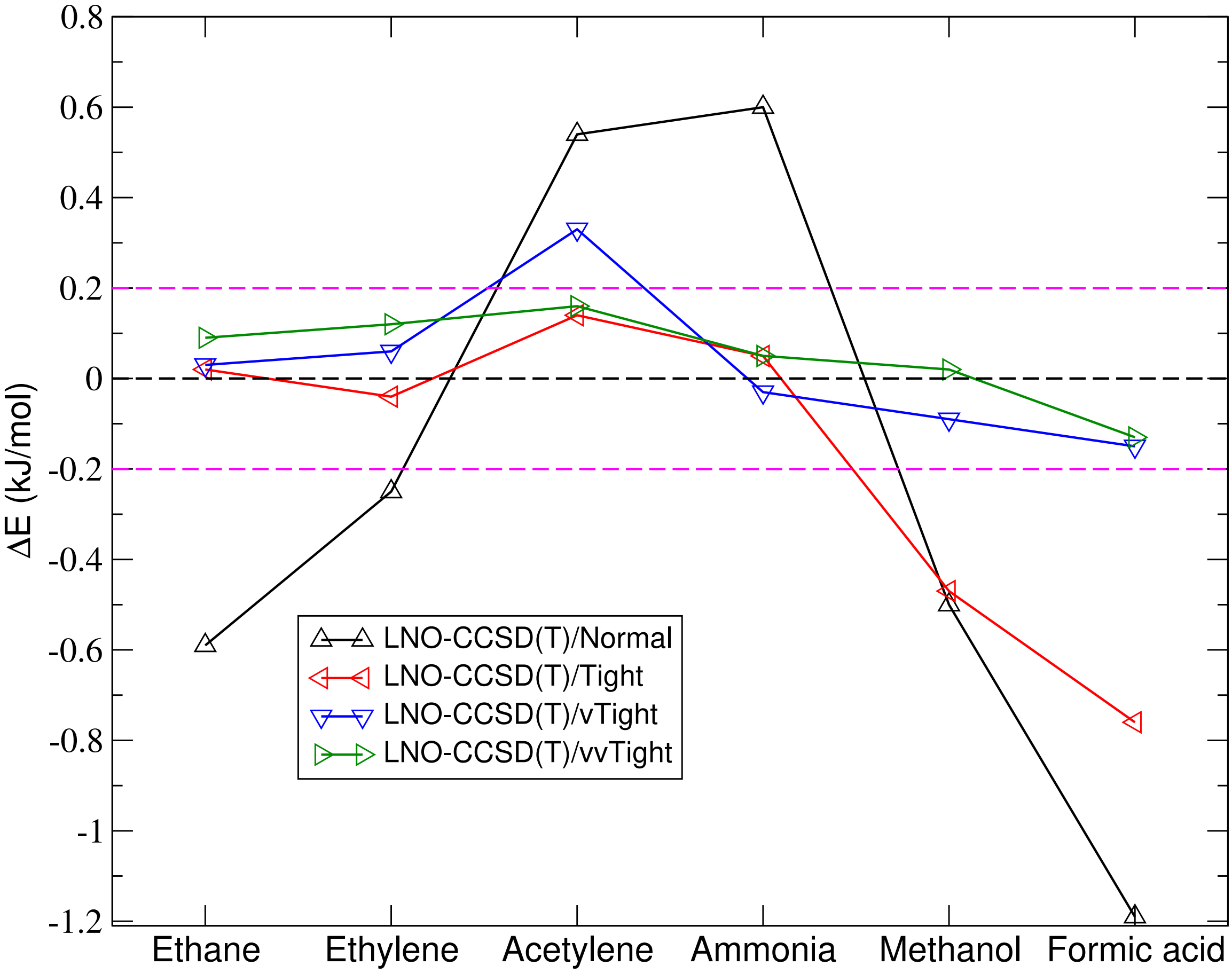

Figure S15: Differences between the LNO-CCSD(T) approximation of nonadditive three-body energies and the canonical reference. The summation is limited to $R = 17$ Å, which excludes the long-range region where the numerical artifacts in the local approximation occur.

8 Accuracy of the multi-level approach

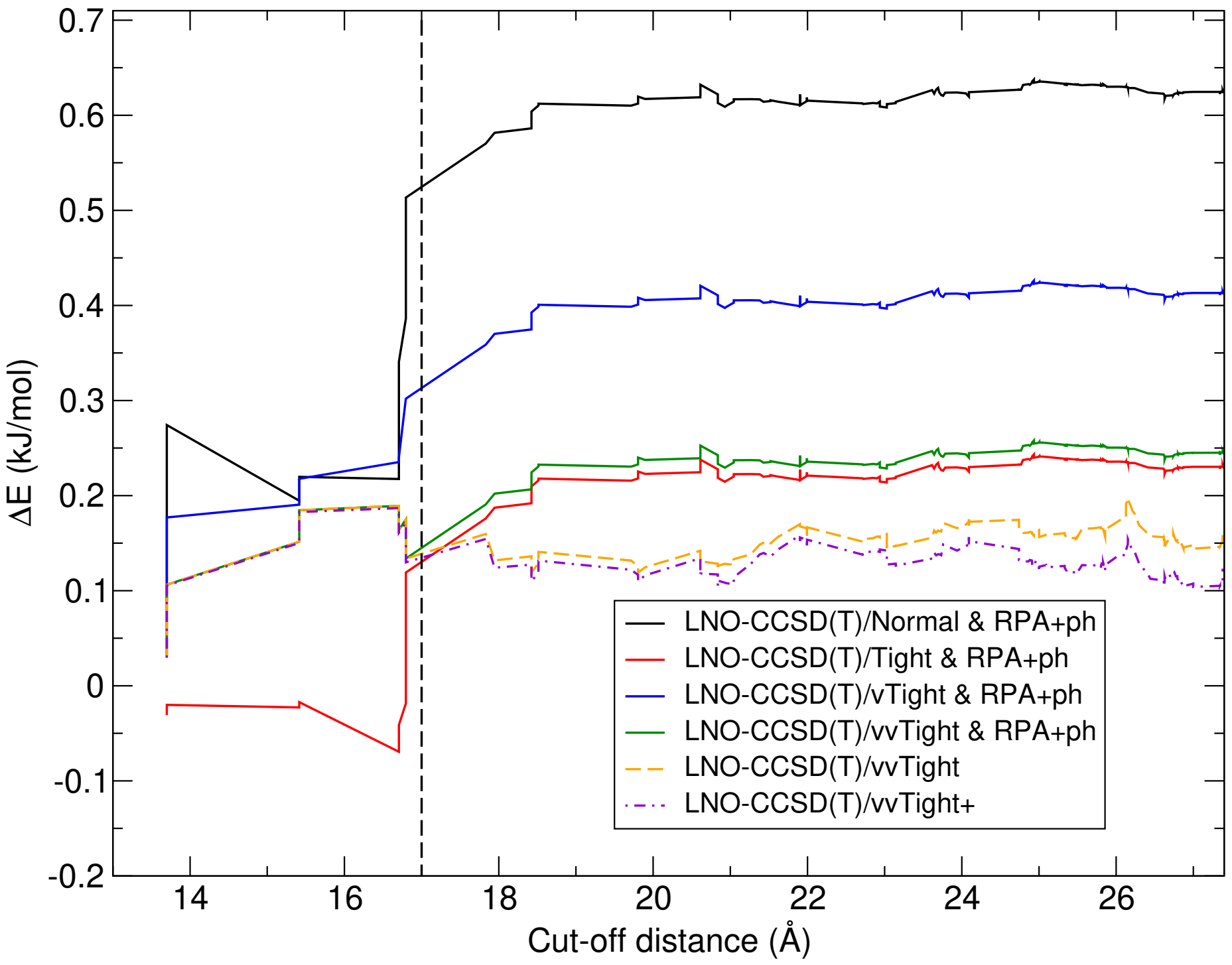

Figure S16: Distance-dependent differences between the multi-level approximations of the nonadditive three-body energy and the canonical CCSD(T) reference for acetylene.

9 CPU timings

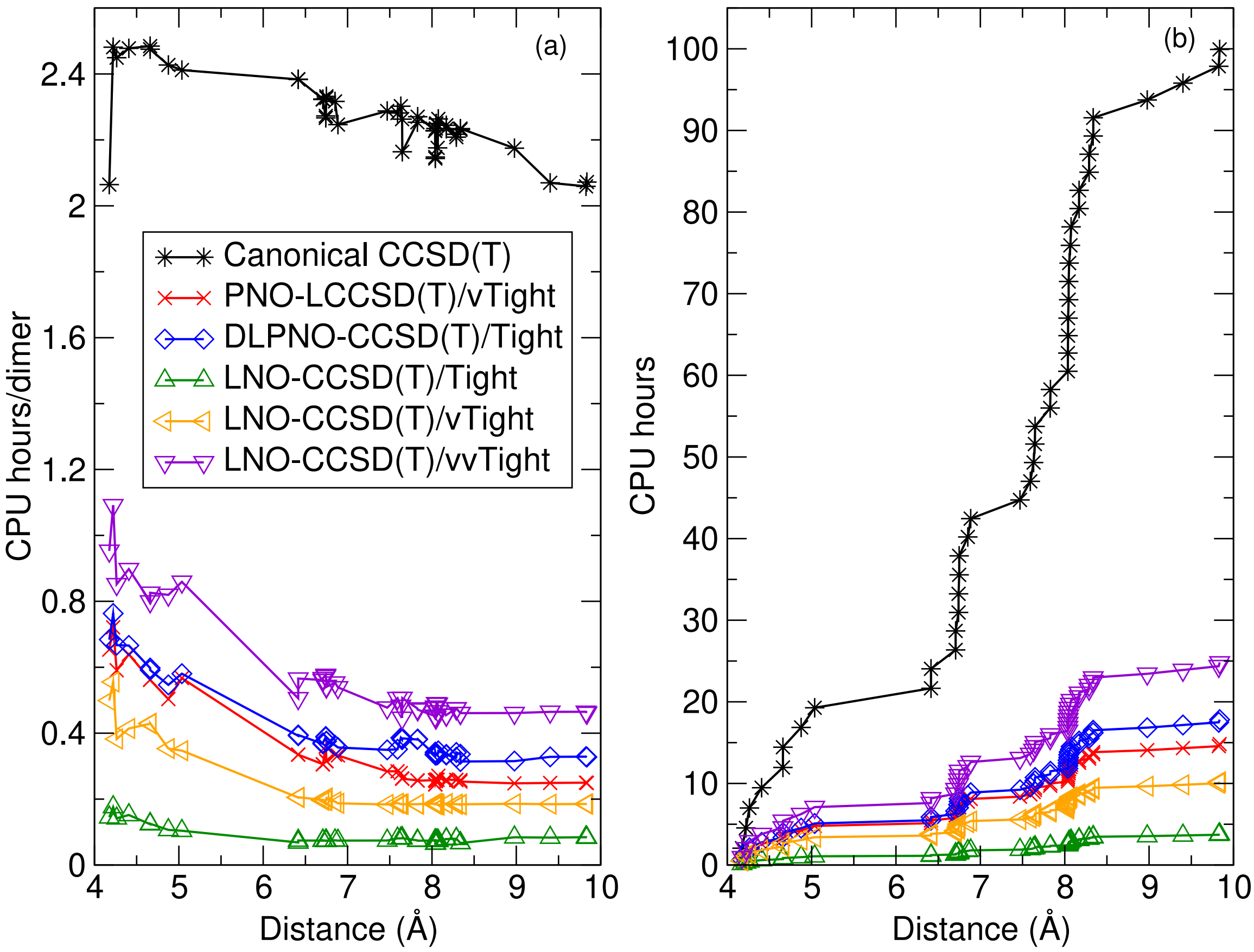

Figure S17: CPU time of individual dimers (a) and cumulative CPU time (b) as a function of intermolecular distance in the two-body expansion of methanol. The data were calculated with AVTZ basis set.

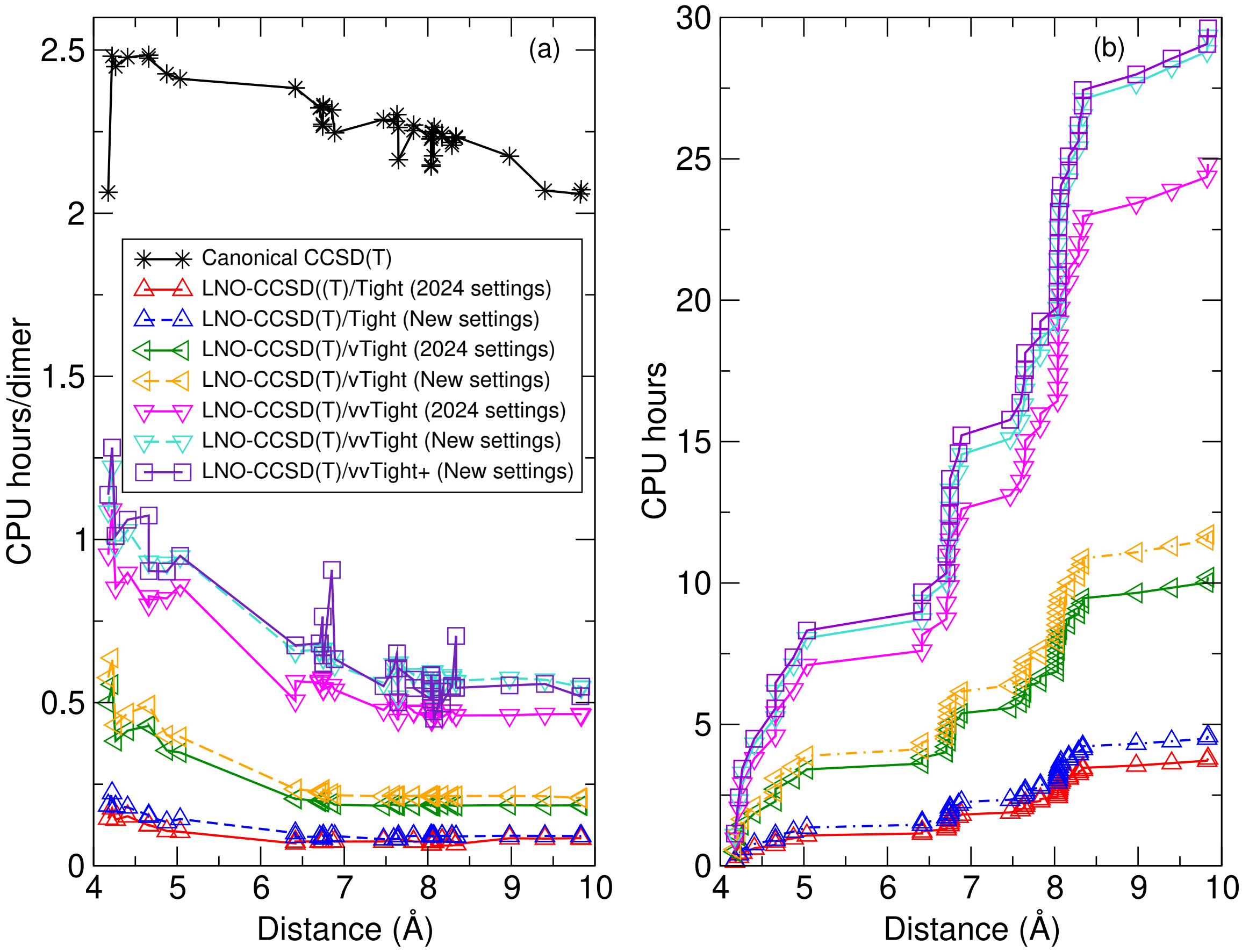

Figure S18: CPU time of individual dimers (a) and cumulative CPU time (b) as a function of intermolecular distance in the two-body expansion of methanol using 2024 and new settings. The data were calculated with AVTZ basis set.

10 Polymorphs of acetylene

Table S12: Two-body, three-body, and beyond-three-body contributions, together with the total binding energies (in kJ/mol), for two forms of acetylene. The beyond-three-body contributions are identical for all methods.

Systems	Methods	Two-body	Three-body	ΔE^{HF}(PBC)	Total
Acetylene (form I)[a]	Canonical CCSD(T)	−25.95	1.15	−0.15	−24.95
	Multi-level(Tight)	−25.51	1.38		−24.28
	Multi-level(vTight)	−26.60	1.57		−25.18
	Multi-level(vvTight)	−26.26	1.40		−25.01
Acetylene (form II)[b]	Canonical CCSD(T)	−27.73	0.97	−0.24	−27.00
	Multi-level(Tight)	−27.51	1.33		−26.42
	Multi-level(vTight)	−28.35	1.03		−27.56
	Multi-level(vvTight)	−28.18	1.35		−27.07

[a] Space group $Pa\bar{3}$ (No. 205).
[b] Space group *Cmce* (No. 64).

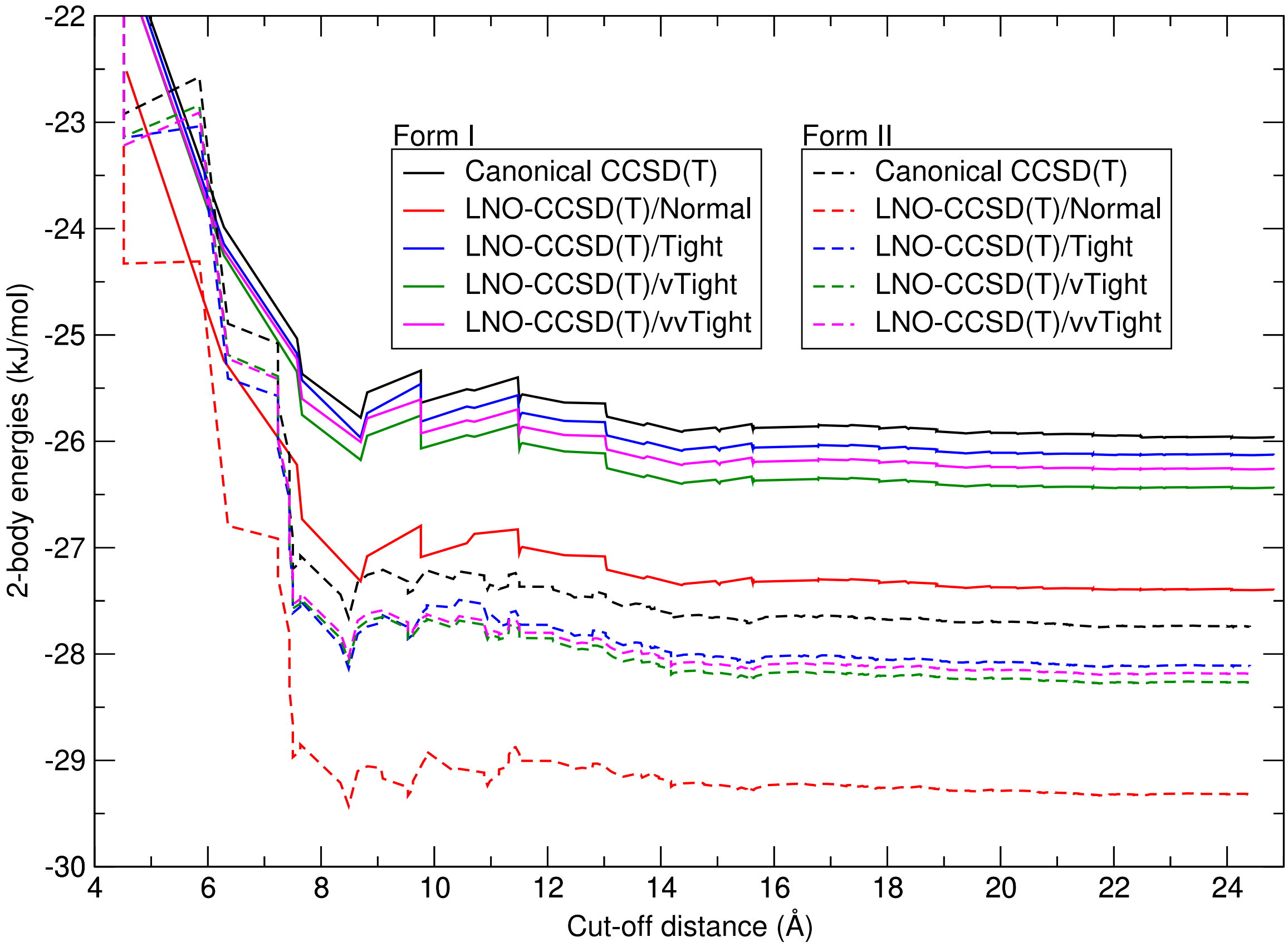

Figure S19: Distance convergence of the two-body energies of acetylene polymorphs.

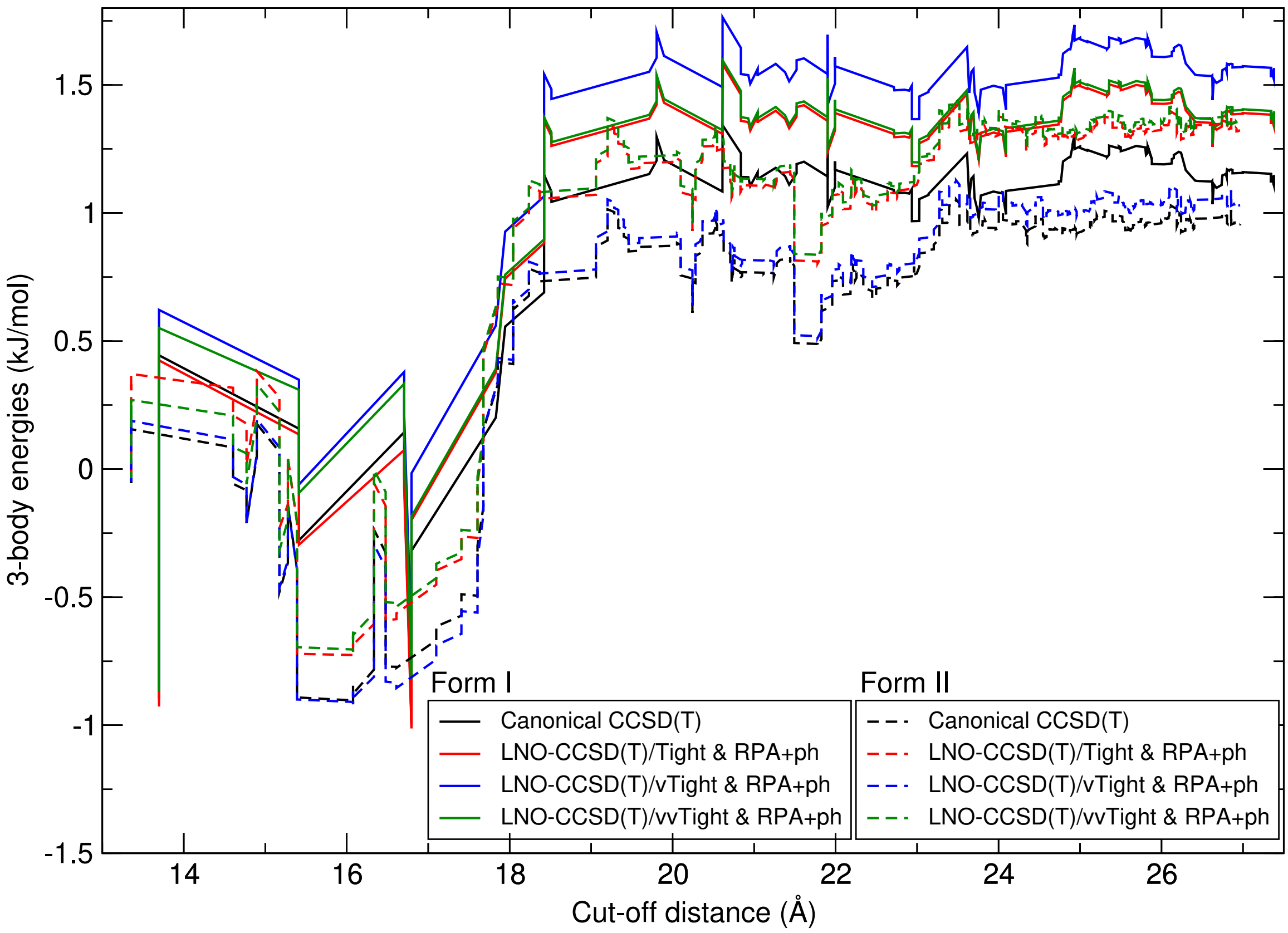

Figure S20: The distance convergence of the three-body energies for two polymorphs of acetylene.

11 DFT results

Table S13: Two-body, three-body, and total binding energies (kJ/mol) obtained from PBE and PBE0 together with the D4 correction. The two-body and three-body DFT energies were computed using the AVQZ and AVTZ basis sets, respectively. The total binding energies were calculated using PBC approach. The reference canonical CCSD(T) data for the two- and three-body interactions and the total binding energy which includes periodic HF calculations were added for comparison and originate from Table 4 of the main text.

Input	Contribution	Ethane	Ethylene	Acetylene	Ammonia	Methanol	Formic acid
PBE	2-body	−28.07	−29.34	−31.71	−45.01	−54.94	−84.02
	3-body	8.13	8.00	6.15	9.43	0.98	7.25
	Total(PBC)	−21.65	−23.16	−26.75	−38.93	−56.36	−84.57
PBE0	2-body	−24.83	−26.64	−30.13	−41.00	−52.06	−84.85
	3-body	5.05	5.09	3.98	5.27	−2.23	2.91
	Total(PBC)	−20.84	−22.74	−26.91	−37.27	−56.06	−87.85
Reference	2-body	−23.86	−25.53	−25.95	−36.61	−49.16	−80.94
	3-body	1.46	1.56	1.15	0.65	−5.93	−1.13
	Total	−22.39	−22.08	−24.95	−35.70	−55.08	−84.96

12 Geometry input file for PBC calculations

```
Ethane solid
1.0
4.2259988785 0.0000000000 0.0000000000
0.0000000000 5.6230001450 0.0000000000
-0.0418251797 0.0000000000 5.8448501436
C H
4 12
Cartesian
4.021313412 0.535489480 5.324639050
0.162859255 5.087510540 0.520210092
2.254946136 3.346988672 3.442635121
1.929226562 2.276009629 2.402213978
0.230831877 0.287659073 4.341575922
3.953340853 5.335340988 1.503273176
1.819427816 3.099159229 4.425698248
2.364743875 2.523840916 1.419150850
0.192004080 1.520774720 5.624243946
3.992167611 4.102224587 0.220605066
1.858255582 4.332274625 3.143030225
2.325917116 1.290724431 2.701819048
2.932632616 0.645360833 5.184784250
1.251540082 4.977638391 0.660064935
3.343626932 3.456860821 3.582489920
0.840545704 2.166138150 2.262359179

Ethane molecule
1.0
25.000 0.000 0.000
0.000 25.000 0.000
0.000 0.000 25.000
```

```
C H
2 6
Cartesian
2.2549500000000000 3.3469899999999999 3.4426399999999999
1.9292300000000000 2.2760099999999999 2.4022100000000002
1.8194300000000001 3.0991599999999999 4.4257000000000000
2.3647399999999998 2.5238399999999999 1.4191499999999999
1.8582600000000000 4.3322700000000003 3.1430300000000000
2.3259200000000000 1.2907200000000001 2.7018200000000001
3.3436300000000001 3.4568599999999998 3.5824900000000000
0.84055000000000002 2.1661400000000000 2.2623600000000001

Ethylene solid
1.0
4.6259989738 0.0000000000 0.0000000000
0.0000000000 6.6199989319 0.0000000000
-0.3113078864 0.0000000000 4.0550669451
C H
4 8
Cartesian
3.780458613 0.362243156 3.887976748
0.534231576 6.257755849 0.167090227
2.691577705 3.672242943 2.194624153
1.623113446 2.947755792 1.860443759
3.698442717 1.149977005 0.484758860
0.616247263 5.470022026 3.570308145
3.084902118 4.459975977 1.542774069
1.229788841 2.160022955 2.512292756
3.248788122 0.199899522 2.944912820
1.065902965 6.420099398 1.110154125
3.223247920 3.509899394 3.137688081
1.091442069 3.110099537 0.917378804
```

```
Ethylene molecule
1.0
25.000 0.000 0.000
0.000 25.000 0.000
0.000 0.000 25.000
C H
2 4
Cartesian
2.6915800000000001 3.6722399999999999 2.1946200000000000
1.6231100000000001 2.9477600000000002 1.8604400000000001
3.0849000000000002 4.4599799999999998 1.5427700000000000
1.2297899999999999 2.1600199999999998 2.5122900000000001
3.2232500000000002 3.5099000000000000 3.1376900000000001
1.0914400000000000 3.1101000000000001 0.917379999999999972
```

```
Acetylene solid (form I)
1.0
6.1050000191 0.0000000000 0.0000000000
0.0000000000 6.1050000191 0.0000000000
0.0000000000 0.0000000000 6.1050000191
C H
8 8
Cartesian
0.348796650 0.348796650 0.348796650
5.756202209 5.756202209 5.756202209
2.703702382 5.756202209 3.401296728
3.401296728 0.348796650 2.703702382
5.756202209 3.401296728 2.703702382
0.348796650 2.703702382 3.401296728
3.401296728 2.703702382 5.756202209
2.703702382 3.401296728 0.348796650
0.969477678 0.969477678 0.969477678
5.135521249 5.135521249 5.135521249
```

```
2.083021240 5.135521249 4.021977688
4.021977688 0.969477678 2.083021240
5.135521249 4.021977688 2.083021240
0.969477678 2.083021240 4.021977688
4.021977688 2.083021240 5.135521249
2.083021240 4.021977688 0.969477678
```

```
Acetylene molecule (form I)
1.0
25.000 0.000 0.000
0.000 25.000 0.000
0.000 0.000 25.000
C H
2 2
Cartesian
3.4013000000000000 2.7037000000000000 5.7561999999999998
2.7037000000000000 3.4013000000000000 6.4538000000000002
4.0219800000000001 2.0830199999999999 5.1355199999999996
2.0830199999999999 4.0219800000000001 7.0744800000000003
```

```
Acetylene solid (form II)
1.0
6.1979990005 0.0000000000 0.0000000000
0.0000000000 6.0229988098 0.0000000000
0.0000000000 0.0000000000 5.5780000687
C H
8 8
Cartesian
0.384960115 0.466012853 0.000000000
5.813039024 5.556985104 0.000000000
3.483960216 2.545486059 0.000000000
2.714038969 3.477512751 0.000000000
0.384960115 3.477512751 2.789000034
```

5.813039024 2.545486059 2.789000034

3.483960216 5.556985104 2.789000034

2.714038969 0.466012853 2.789000034

1.071152791 1.296126500 0.000000000

5.126846209 4.726872400 0.000000000

4.170151922 1.715372457 0.000000000

2.027846155 4.307626533 0.000000000

1.071152791 4.307626533 2.789000034

5.126846209 1.715372457 2.789000034

4.170151922 4.726872400 2.789000034

2.027846155 1.296126500 2.789000034

Acetylene molecule (form II)

1.0

25.000 0.000 0.000

0.000 25.000 0.000

0.000 0.000 25.000

C H

2 2

Cartesian

3.4839601519999999 5.5569849009999999 2.7890000339999999

2.7140390870000002 6.4890116747999995 2.7890000339999999

4.1701517109999999 4.7268724439999996 2.7890000339999999

2.0278460979999999 7.3191252948000001 2.7890000339999999

Ammonia solid

1.0

5.1304998398 0.0000000000 0.0000000000

0.0000000000 5.1304998398 0.0000000000

0.0000000000 0.0000000000 5.1304998398

N H

4 12

Cartesian

1.080996343 1.080996343 1.080996343

3.646246110 1.484253576 4.049503649

4.049503649 3.646246110 1.484253576

1.484253576 4.049503649 3.646246110

1.892641396 1.370356540 0.594624944

1.370356540 0.594624944 1.892641396

0.594624944 1.892641396 1.370356540

4.457891316 1.194893380 4.535875011

3.935606307 1.970624938 3.237858444

3.159874749 0.672608524 3.760143453

3.760143453 3.159874749 0.672608524

4.535875011 4.457891316 1.194893380

1.970624938 3.237858444 3.935606307

0.672608524 3.760143453 3.159874749

1.194893380 4.535875011 4.457891316

3.237858444 3.935606307 1.970624938

Ammonia molecule

1.0

25.000 0.000 0.000

0.000 25.000 0.000

0.000 0.000 25.000

N H

1 3

Cartesian

1.08099635 1.08099635 1.08099635

1.89264145 1.37035655 0.59462495

1.37035655 0.59462495 1.89264145

0.59462495 1.89264145 1.37035655

Methanol solid

1.0

4.8727998734 0.0000000000 0.0000000000

0.0000000000 4.6410999298 0.0000000000

0.0000000000 0.0000000000 8.8670997620

C O H

4 4 16

Cartesian

0.900006123 2.354893994 0.935479005

1.536393741 2.286206074 5.369028754

3.972793677 0.034344029 3.498071008

3.336406133 4.606755624 7.931620889

2.168396054 2.204522439 1.526027804

0.268004028 2.436577352 5.959577817

2.704403965 4.525072542 2.907521945

4.604795845 0.116027388 7.341071826

2.407163078 3.032030525 2.053620318

0.029236861 1.609069405 6.487170067

2.465636940 0.711480560 2.379929695

4.843563160 3.929619369 6.813479576

0.732869090 1.450807777 0.352023874

1.703530919 3.190292015 4.785574019

4.139930856 3.771357880 4.081525743

3.169268954 0.869742603 8.515075624

0.140823913 2.421261948 1.668788148

2.295576033 2.219837844 6.102338029

4.731976115 0.100711983 2.764761733

2.577223986 4.540388224 7.198311614

0.895133314 3.168943009 0.243845242

1.541266623 1.472156921 4.677394892

3.977666705 0.848393044 4.189704342

3.331533105 3.792706886 8.623254751

Methanol molecule

1.0

25.000 0.000 0.000

0.000 25.000 0.000

0.000 0.000 25.000

C O H

1 1 4

Cartesian

0.90001 2.35489 0.93548

2.16840 2.20452 1.52603

0.14082 2.42126 1.66879

2.40716 3.03203 2.05362

0.73287 1.45081 0.35202

0.89513 3.16894 0.24385

Formic acid solid

1.0

10.2410001755 0.0000000000 0.0000000000

0.0000000000 3.5439999104 0.0000000000

0.0000000000 0.0000000000 5.3559999466

C O H

4 8 8

Cartesian

1.624567383 1.110862472 0.889070737

8.616432945 2.433137438 3.567070790

6.745067319 0.661137483 0.889070737

3.495932552 2.882862427 3.567070790

0.877883360 0.501879651 0.001412370

9.363116586 3.042120048 2.679412621

5.998383066 1.270120304 0.001412370

4.242616804 2.273879606 2.679412621

2.856646179 1.222254741 0.782757806

7.384353997 2.321745064 3.460757938

7.977146266 0.549745161 0.782757806

2.263853909 2.994254802 3.460757938

1.458201038 0.118216010 4.580264997

8.782799290 3.425783887 1.902265023

6.578701583 1.653784037 4.580264997

3.662299203 1.890215978 1.902265023

1.061152907 1.527043365 1.741127225

9.179847345 2.016956335 4.419127677

6.181652918 0.244956538 1.741127225

4.059347257 3.299043531 4.419127677

Formic acid molecule

1.0

25.000 0.000 0.000

0.000 25.000 0.000

0.000 0.000 25.000

C O H

1 2 2

Cartesian

1.6245673672185279 1.1108624528748128 0.88907076421716558

0.87788333208995550 0.50187962672739717 0.0014123699298367869

2.8566461616491212 1.2222547708334826 0.78275777389053114

1.4582009671123854 0.11821600622660995 -0.77573494655506092

1.0611529418512515 1.5270433945612862 1.7411272199175354
